# A Blockchain-Based Dual-Track Mechanism for Trusted Circulation of Food Safety Detection Data and Batch-Level Risk Control: An Aflatoxin B1 Case Study

**DOI:** 10.3390/foods15173055

**Published:** 2026-08-28

**Authors:** Mingyang Chen, Zhiyao Zhao, Jiping Xu, Jiabin Yu, Xiaoyu Cui, Xin Zhang

**Affiliations:** 1 School of Computer and Artificial Intelligence, Beijing Technology and Business University, Beijing 100048, China; 2531062172@st.btbu.edu.cn (M.C.); zhaozy@btbu.edu.cn (Z.Z.); xujiping@139.com (J.X.); yujiabin@th.btbu.edu.cn (J.Y.); xiaoyucui@btbu.edu.cn (X.C.); 2 Beijing Key Laboratory of Big Data Technology for Food Safety, Beijing Technology and Business University, Beijing 100048, China

**Keywords:** food safety, aflatoxin B1, detection-data management, blockchain, IPFS, multi-oracle, smart contract, batch-level risk control

## Abstract

Food-safety systems increasingly need to manage large detection files and externally generated analytical results across multiple organizations while linking these records to batch-level control actions. This study proposes a blockchain-based dual-track mechanism for trusted circulation of food-safety detection data and batch-level risk control, using aflatoxin B1 (AFB1) as the empirical case. High-dimensional files are stored in InterPlanetary File System (IPFS) and anchored on-chain by content identifiers (CIDs); three authorized oracles use two-of-three matching of detection values and evidence hashes before contract-based state determination. A stage–device–operation inverted index identifies associated batches, while signed second-track confirmations drive GREEN/YELLOW/RED state transitions. Experiments on a four-node Quorum Byzantine Fault Tolerance (QBFT) network used 57 independent HyperPistachio samples with 86.625-mebibyte (MiB) band-interleaved-by-line (BIL) files. Real-file access was successfully completed, single-oracle failures were tolerated when two consistent oracle reports remained, and associated-batch query latency increased only from 13.06 to 16.78 ms as fanout rose from 1 to 40. A 115.15 min sustained run maintained consistent states across all four nodes. The study manages externally supplied AFB1 results rather than evaluating analytical AFB1 detection accuracy, and its experimental validation is limited to the AFB1 case.

## 1. Introduction

### 1.1. Research Background

Food safety management requires continuous control of contaminant-related risks across production, processing, storage, transportation, and sales [1]. Digital traceability and smart-contract technologies are increasingly being introduced to support trusted information management across these stages [2]. Visual analytics and predictive digital technologies are further extending food-safety supervision from retrospective inspection toward data-supported monitoring and decision-making [3,4]. Meanwhile, artificial intelligence and Internet of Things (IoT)-enabled systems are increasing the diversity and volume of data generated during food testing and monitoring [5,6]. Spectroscopic detection and integrated digital traceability can generate high-dimensional files, structured analytical results, device records, and other heterogeneous information that must subsequently be stored, transferred, and associated with food batches [7,8].

However, incorporating such detection data into cross-organizational risk-control workflows remains challenging. Blockchain- and IoT-based food-safety architectures provide mechanisms for trusted recording and information sharing [9,10], but detection results originating outside the blockchain still depend on the integrity and availability of their external data sources [11,12]. Existing smart-contract and traceability systems can record supply-chain events and enforce predefined business rules [13,14], yet they generally focus on provenance and circulation records rather than linking high-dimensional detection evidence to associated-batch suspension and subsequent confirmation [15].

Accordingly, this study focuses on three connected data-management problems: low-cost access to high-dimensional detection files, trusted admission of externally supplied analytical results, and controlled localization of batches associated with a detected risk source. To address these problems, a blockchain-based dual-track mechanism is developed by integrating InterPlanetary File System (IPFS) storage with on-chain content identifier (CID) anchoring, multi-oracle arbitration, a stage–device–operation inverted index, and signature-based second-track confirmation. The resulting workflow connects detection evidence, batch-state determination, associated-batch locking, and confirmatory release or blocking. The mechanism is formulated for food-safety detection-data management, while its empirical validation in this study is specifically limited to aflatoxin B1 (AFB1) data from the HyperPistachio case.

### 1.2. Related Work

#### 1.2.1. Blockchain and Food-Safety Data Management

Food supply chains involve multiple stakeholders, dispersed data sources, long circulation processes, and difficulties in responsibility attribution. Owing to its immutability, traceability, and multiparty sharing capabilities, blockchain has been applied to trusted food supply-chain data management to improve the transparency of food origin, circulation, quality and safety, and sustainability information. Tang et al. reported that integrating blockchain with the Internet of Things can improve food safety, transparency, and traceability in agri-food supply chains, although infrastructure, cost, and regulatory adaptation remain limiting factors [16]. Rajput et al. argued that distributed records, smart contracts, and transparent information sharing can enhance traceability, accountability, and sustainability in food supply chains [17]. From a food-waste governance perspective, Silayo et al. found that blockchain can strengthen supply-chain transparency and accountability but remains constrained by digital literacy, infrastructure, and data-governance capacity [18]. Soe et al. developed a blockchain system for tracing the carbon footprint of organic food to improve certification-data transparency and multi-stakeholder collaboration [19]. Karkanias et al. noted that blockchain can enhance product-level traceability, data integrity, and sustainability-claim verification in agri-food systems, although heterogeneous standards, limited interoperability, and consortium governance remain challenges [20].

Overall, blockchain has been widely applied to food traceability, quality certification, supply-chain transparency, and sustainability management, but most existing systems focus on provenance and circulation records rather than the downstream management of analytical detection evidence. High-dimensional detection files and externally generated measurement results impose different requirements because they must remain verifiably linked to the batch state and to subsequent control decisions. Therefore, the gap addressed in this study is not general food traceability itself, but the integration of detection-file references, externally supplied analytical results, and batch-control actions within an auditable workflow.

#### 1.2.2. Off-Chain Storage, Oracles, and Smart Contracts

In digital food supply-chain management, blockchain provides immutable and multiparty-shared records, but food testing, dynamic monitoring, and sensor data are often diverse in format, large in volume, and dispersed across sources. Storing all such data directly on-chain increases storage pressure and execution costs. Accordingly, research has shifted toward collaborative models integrating off-chain storage, on-chain hash anchoring, trusted data access, and automated smart contract execution. Yu et al. indicated that blockchain can be combined with radio-frequency identification (RFID), sensors, and intelligent detection technologies to support real-time monitoring, quality traceability, and trusted information sharing in fresh-food supply chains [21]. Chen et al. proposed the LAP-chain architecture, which integrates redactable blockchain with IPFS by storing time-sensitive agri-food traceability data in IPFS while retaining operation records and hash indexes on-chain, thereby reducing storage pressure while preserving traceability [22]. These studies suggest that large-scale data such as food detection files, sensor logs, and image features are more appropriately managed through off-chain storage and on-chain anchoring.

Beyond storage, the trustworthiness of off-chain data sources and contract-triggering mechanisms is also critical. Liu et al. proposed a full-process food traceability model based on a master–slave multi-chain architecture and trusted transmission protocol, using active RFID collection, trusted transmission, smart contracts, and access control to improve the trustworthiness of data acquisition, transmission, and querying [23]. Manoj et al. noted that blockchain is a closed and deterministic state system in which smart contracts cannot directly access real-world data, requiring oracles to connect off-chain data with on-chain contracts [24]. The Edge–Cloud–Blockchain–Terminal framework proposed by Huang et al. further showed that blockchain can serve as trusted infrastructure linking edge intelligence, cloud analytics, and user terminals [25]. These studies establish a foundation for off-chain storage, trusted acquisition, oracle access, and multilayer architectural collaboration. However, two issues remain insufficiently integrated: the dependence of state-changing decisions on a single oracle service, and the lack of a continuous workflow linking verifiable detection evidence to associated-batch suspension, authenticated confirmation, and final state updating.

#### 1.2.3. AFB1 Detection Data and Digital Batch-Risk Control

Rapid and non-destructive food analysis increasingly relies on spectroscopy and artificial intelligence to extract contamination-related information from complex sample data [26,27]. For AFB1, recent studies have investigated rapid immunoassay and hyperspectral approaches for detection or quantitative analysis [28,29], while deep-learning-assisted hyperspectral models further demonstrate the increasing data and model complexity of AFB1 analysis [30]. These developments mean that food-safety information systems may need to manage not only final analytical values but also high-dimensional source files and the digital evidence associated with those values.

Analytical detection alone, however, does not determine how a reported result should be connected to downstream batch-control actions. Downstream food-safety management further requires structured relationships among analytical results, regulatory entities, standards, and related management information [31], while conventional digital traceability mainly focuses on tracking product and supply-chain events [32]. A gap therefore remains between analytical result generation and an auditable control workflow that connects “detection evidence—reported result—batch-state determination—associated-batch control—confirmatory decision”. The present study addresses this downstream management problem rather than proposing a new AFB1 detection method. The HyperPistachio dataset [33] is used as the empirical case to evaluate whether real hyperspectral files and externally supplied AFB1 concentration labels can participate in this workflow.

Based on the above gap, this study addresses the following research questions (RQs):

RQ1: How can high-dimensional detection files and externally supplied AFB1 results be incorporated into an on-chain/off-chain workflow without directly storing the original files on-chain?

RQ2: How can multi-oracle arbitration reduce dependence on a single oracle service, and what failure boundary remains under a two-of-three mechanism?

RQ3: How can associated batches be selectively locked and subsequently confirmed while limiting over-blocking and maintaining manageable execution overhead?

### 1.3. Contributions of This Study

The contribution of this study lies in the design and validation of a detection-result-driven batch-control workflow rather than in the isolated use of blockchain, IPFS, or digital signatures. The main contributions are as follows.

First, a dual-track state-control mechanism is developed to connect detection evidence, risk-source identification, associated-batch suspension, confirmatory re-examination, and GREEN/YELLOW/RED state transitions within an auditable workflow.

Second, an evidence-bound multi-oracle mechanism requires a two-of-three exact match of valueScaled and evidenceHash before an off-chain result can drive state control, reducing dependence on a single oracle while retaining an explicit quorum-failure boundary.

Third, a stage–device–operation inverted index is coupled with the YELLOW pending-confirmation state, distinguishing association with a risk source from confirmed risk and thereby reducing unnecessary locking compared with coarse-grained strategies.

Fourth, the mechanism is validated using real HyperPistachio AFB1 files, multi-oracle fault scenarios, associated-batch scaling tests, repeated end-to-end dual-track execution with different second-track confirmation orders, and sustained Quorum Byzantine Fault Tolerance (QBFT) operation. The empirical validation concerns management of externally supplied AFB1 results rather than analytical AFB1 detection accuracy.

## 2. Dual-Track Risk-Control Mechanism and Methodology

### 2.1. Overall Logical Framework and Dual-Track State Transition Mechanism

Food-safety detection data managed by the proposed framework may originate from dispersed sources and vary substantially in format and volume. Such data can include high-dimensional preliminary-screening files and smaller structured confirmatory records. The framework is formulated at the data-management level; however, its empirical validation in this study is limited to AFB1-related hyperspectral files and externally supplied AFB1 concentration labels. The framework therefore does not imply that analytical performance has been validated for other contaminants.

Food supply-chain risks may also extend to batches sharing equipment, storage locations, vehicles, or operational units. Blocking only the problematic batch may overlook associated risks, whereas broadly locking all batches at the same node or enterprise may cause over-blocking. Blockchain is therefore used not only for data notarization but also as a collaborative execution layer for trusted data access, multi-oracle result arbitration, associated-batch locking, and state updating.

**Figure 1:**
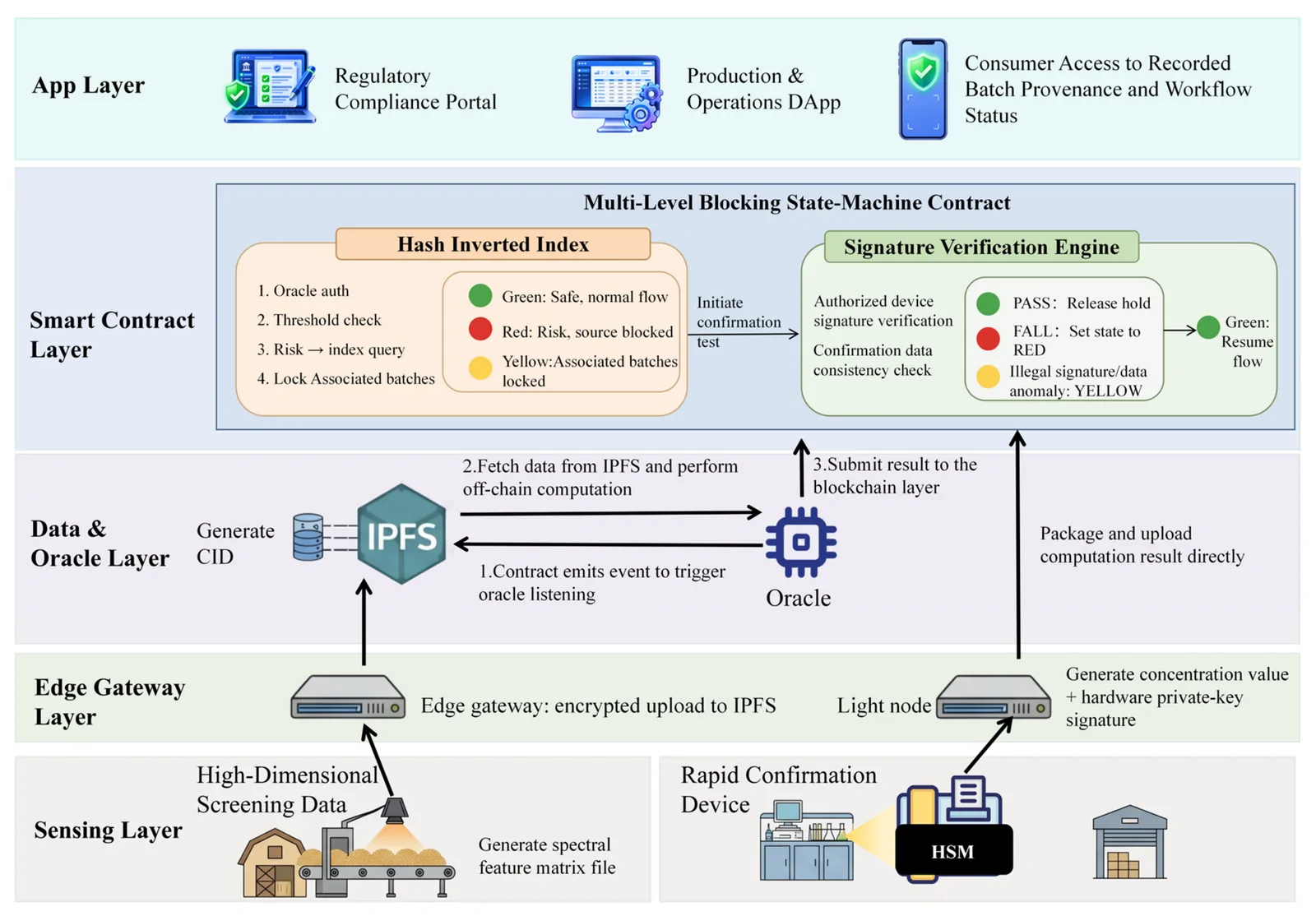
Logical framework for trusted circulation of food-safety detection data and batch-level risk control. DApp, decentralized application; HSM, hardware security module; IPFS, InterPlanetary File System; CID, content identifier. GREEN, YELLOW, and RED denote normal-flow, pending-confirmation, and risk-blocked workflow states, respectively.

Based on these requirements, this study constructs a blockchain-based dual-track architecture for trusted circulation of food-safety detection data and batch-level risk control. The architecture follows the workflow of “high-dimensional detection-data access—multi-oracle result arbitration—contract-based state determination—associated-batch locking—second-track confirmation—state updating” and comprises five layers. The sensing layer provides preliminary-screening and confirmatory data; the edge gateway uploads detection files and structured records; the data and oracle layer connect off-chain evidence with on-chain rules; the smart-contract layer performs result application, state updating, associated-batch locking, and signature verification; and the application layer supports batch-state querying, risk tracing, circulation control, and traceability. In this framework, consumer-facing traceability is limited to access to recorded batch provenance and the current system state generated by the predefined workflow. It does not constitute analytical verification of the absence of AFB1, regulatory compliance, or an overall food-safety certification. The overall architecture is shown in Figure 1.

The left track provides trusted access to high-dimensional detection files and applies externally supplied analytical results to source-batch state determination, whereas the second track confirms associated batches and determines their release or blocking. The two tracks are connected through a smart contract state machine. A batch classified as Risk by the left track is updated to RED, and the hash-based inverted index locks associated batches in YELLOW. A passed second-track confirmation restores the batch to GREEN, a failed confirmation updates it to RED, and an ILLEGAL confirmation leaves it in YELLOW. Figure 1 summarizes the resulting batch-level risk-control loop linking detection files, detection values, device–operation relationships, and confirmatory conclusions.

**Figure 2:**
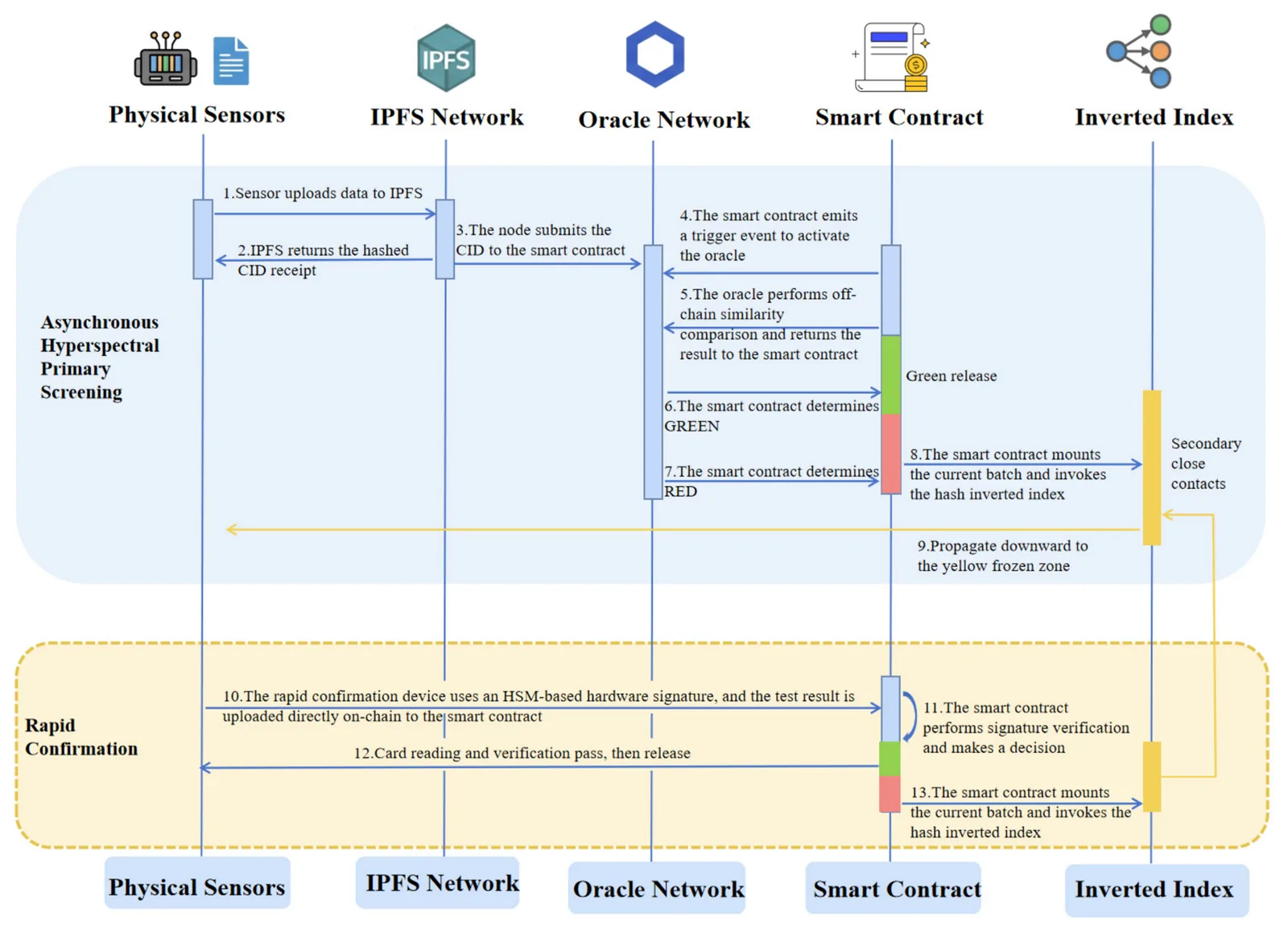
Blockchain-based on-chain/off-chain collaborative interaction sequence diagram.

Figure 2 presents the on-chain/off-chain interaction sequence of the dual-track mechanism. During left-track, the edge node uploads a detection file to IPFS and submits its CID on-chain. The authorized oracle services independently retrieve the CID-addressed evidence and submit valueScaled and evidenceHash. The multi-oracle controller forms an applicable result only when at least two reports contain an exactly matching value–evidence pair, after which the dual-track contract performs threshold-based state determination. A RED source batch triggers associated-batch locking in YELLOW through the device-use index. YELLOW batches then enter second-track confirmation, where signature, authorization, result-consistency, and duplicate-submission checks determine whether they are restored to GREEN, updated to RED, or remain YELLOW.

The batch states and their transition rules are defined in Table 1 and Table 2.

To describe the batch state transition process, the state set is defined as

(1) S={GREEN,YELLOW,RED}

The event set is defined as

(2) $$E=\{e_{\mathrm{safe}},e_{\mathrm{risk}},e_{\mathrm{lock}},e_{\mathrm{pass}},e_{\mathrm{fail}},e_{\mathrm{invalid}}\}$$

where $e_{\mathrm{safe}}$ denotes that the left-track result does not trigger the configured risk-control threshold; $e_{\mathrm{risk}}$ denotes that, after oracle-based off-chain computation, the smart contract determines the left-track detection result to be risky according to the on-chain threshold; $e_{\mathrm{lock}}$ indicates that the batch is matched by the hash-based inverted index; $e_{\mathrm{pass}}$ indicates that the second-track confirmation result is qualified and the signature verification is passed; $e_{\mathrm{fail}}$ indicates that the second-track confirmation result is unqualified while the signature verification is passed; and $e_{\mathrm{invalid}}$ indicates an illegal confirmation submission, including an illegal signature, an unauthorized device, inconsistent fields, or repeated submission. The batch state transition rules are shown in Table 2.

The state transition function can be expressed as follows:

(3) $$S_{t+1}=f\left(S_{t},e\right)$$

where $S_{t}$ denotes the current batch state, e denotes the triggering event, and $S_{t+1}$ denotes the new state after the event occurs. The core transition rules are expressed as

(4) $$S_{t+1}=\left\{\begin{array}{cc} \mathrm{GREEN}, & S_{t}=\mathrm{GREEN}\wedge e=e_{\mathrm{safe}}\\ \mathrm{RED}, & S_{t}=\mathrm{GREEN}\wedge e=e_{\mathrm{risk}}\\ \mathrm{YELLOW}, & S_{t}=\mathrm{GREEN}\wedge e=e_{\mathrm{lock}}\\ \mathrm{GREEN}, & S_{t}=\mathrm{YELLOW}\wedge e=e_{\mathrm{pass}}\\ \mathrm{RED}, & S_{t}=\mathrm{YELLOW}\wedge e=e_{\mathrm{fail}}\\ \mathrm{YELLOW}, & S_{t}=\mathrm{YELLOW}\wedge e=e_{\mathrm{invalid}}\\ \mathrm{RED}, & S_{t}=\mathrm{RED}\\ S_{t}, & \mathrm{otherwise} \end{array}\right.$$

Specifically, the off-chain storage and multi-oracle arbitration process is described in Section 2.2, the associated-batch locking mechanism in Section 2.3, and second-track signature-based confirmation in Section 2.4.

### 2.2. IPFS- and Multi-Oracle-Assisted Off-Chain Data Processing Mechanism

High-dimensional analytical files are unsuitable for routine direct on-chain storage because their size increases Gas consumption and transaction-capacity requirements. The proposed mechanism therefore stores left-track files in IPFS and anchors their CIDs on-chain for verifiable access. After a CID-submission event is emitted, three independently authorized oracle services separately retrieve the same CID-addressed evidence and obtain the corresponding externally supplied analytical value and evidence hash. These services provide independently authorized reporting paths rather than independent analytical measurements. A result is admitted to the dual-track state-control contract only after at least two of the three authorized oracles submit an exactly matching (valueScaled, evidenceHash) pair. The smart contract then performs threshold-based state determination using the arbitrated result. Thus, the multi-oracle layer reduces dependence on a single authorized oracle service, although it does not independently establish the analytical correctness of the upstream measurement.

Let $b_{i}$ denote the i-th food batch, and let $F_{i}$ denote its left-track preliminary screening detection file. The system first uploads the detection file to IPFS, where a unique content identifier is generated according to the file content:

(5) $${\mathrm{CID}}_{i}=\mathrm{IPFSHash}(F_{i})$$

where ${\mathrm{CID}}_{i}$ denotes the content-addressed identifier of $F_{i}$. Binding ${\mathrm{CID}}_{i}$ to $b_{i}$ enables integrity verification and traceable access without storing the original detection file directly on-chain.

After obtaining ${\mathrm{CID}}_{i}$, the edge node calls submitCID() to submit the batch identifier and CID. The contract records their binding and emits a CIDSubmitted event:

(6) $$E_{\mathrm{CID}}=(b_{i},{\mathrm{CID}}_{i},h_{i},\theta_{i},t_{i},a_{s})$$

where $h_{i}$, $\theta_{i}$, $t_{i}$, and $a_{s}$ denote the file hash, on-chain determination threshold, submission time, and submitting address, respectively. The CIDSubmitted event is then monitored independently by the three authorized oracles $O_{k}$, k∈{1,2,3}.

After detecting the CIDSubmitted event, each authorized oracle $O_{k}$ separately retrieves the CID-addressed file $F_{i}$ and reads the corresponding externally supplied analytical value:

(7) $$v_{i,k}=f_{k}(F_{i}),\;\;k\in \{1,2,3\}$$

where $v_{i,k}$ denotes the value obtained by oracle $O_{k}$, and $f_{k}(\cdot )$ denotes its off-chain retrieval and value-mapping operation. In the AFB1 case, this operation reads the dataset-provided concentration label rather than executing a newly trained AFB1 detection model.

To support integer-based smart-contract processing, each oracle converts $v_{i,k}$ into the integer representation used by the smart contract:

(8) $$v_{i,k}^{\mathrm{scaled}}=\mathrm{Scale}(v_{i,k})$$

where $v_{i,k}^{\mathrm{scaled}}$ denotes the scaled value submitted by oracle $O_{k}$ for integer-based contract processing.

Each oracle then submits its scaled value together with the evidence hash using its authorized identity. For batch $b_{i}$, the report submitted by $O_{k}$ is represented as

(9) $$\left(b_{i},v_{i,k}^{\mathrm{scaled}},h_{i,k},t_{i,k},O_{k}\right)$$

where $h_{i,k}$ and $t_{i,k}$ denote the evidence hash and submission time reported by oracle $O_{k}$, respectively. For a correct report, the submitted evidence hash is consistent with the evidence bound to $F_{i}$.

The multi-oracle controller verifies the oracle identities and compares the submitted value–evidence pairs. An applicable result is formed only when at least two authorized reports contain exactly the same pair. Using the existing $v_{i,k}^{\mathrm{scaled}}$ and $h_{i}$ to denote the pair admitted for batch $b_{i}$, the two-of-three condition is

(10) $$\sum_{k=1}^{3}1\left[\left(v_{i,k}^{\mathrm{scaled}},h_{i,k})=(v_{i}^{\mathrm{scaled}},h_{i}\right)\right]\geq 2$$

where $1\left[\cdot \right]$ is the indicator function. If this condition is satisfied, $v_{i,k}^{\mathrm{scaled}}$ becomes the arbitrated value passed to the subsequent threshold rule; otherwise, the oracle task remains PENDING, and no new batch-state transition is executed.

Once a two-of-three match is formed, the admitted $v_{i,k}^{\mathrm{scaled}}$ is evaluated using the existing on-chain threshold rule:

(11) $$R_{i}=\left\{\begin{array}{cc} \mathrm{Safe}, & v_{i}^{\mathrm{scaled}}\leq \theta_{i}^{\mathrm{scaled}}\\ \mathrm{Risk}, & v_{i}^{\mathrm{scaled}}>\theta_{i}^{\mathrm{scaled}} \end{array}\right.$$

where $\theta_{i}^{\mathrm{scaled}}$ denotes the scaled threshold recorded in the smart contract, and $R_{i}$ denotes the Safe/Risk determination.

The accepted determination result is then mapped to the existing batch-state definition:

(12) $$\mathrm{State}(b_{i})=\left\{\begin{array}{cc} \mathrm{GREEN}, & R_{i}=\mathrm{Safe}\\ \mathrm{RED}, & R_{i}=\mathrm{Risk} \end{array}\right.$$

When $R_{i}=\mathrm{Safe}$, the smart contract updates or maintains the batch state as GREEN. When $R_{i}=\mathrm{Risk}$, the smart contract updates the current batch to RED and further triggers the hash-based inverted-index module to retrieve and lock associated batches that have shared-equipment relationships with the identified risk-source batch.

The oracle-processing time is decomposed into event listening, IPFS retrieval, off-chain value processing, and on-chain report/application time:

(13) $$T_{\mathrm{oracle}}=T_{\mathrm{listen}}+T_{\mathrm{fetch}}+T_{\mathrm{compute}}+T_{\mathrm{callback}}$$

where $T_{\mathrm{listen}}$, $T_{\mathrm{fetch}}$, $T_{\mathrm{compute}}$, and $T_{\mathrm{callback}}$ denote event detection, CID-based file retrieval, off-chain value processing, and on-chain report confirmation and result application, respectively. In the three-oracle mode, the oracle services execute concurrently; therefore, $T_{\mathrm{oracle}}$ represents the critical path up to the matching report that completes the two-of-three quorum rather than the sum of the execution times of all three oracles.

**Figure 3:**
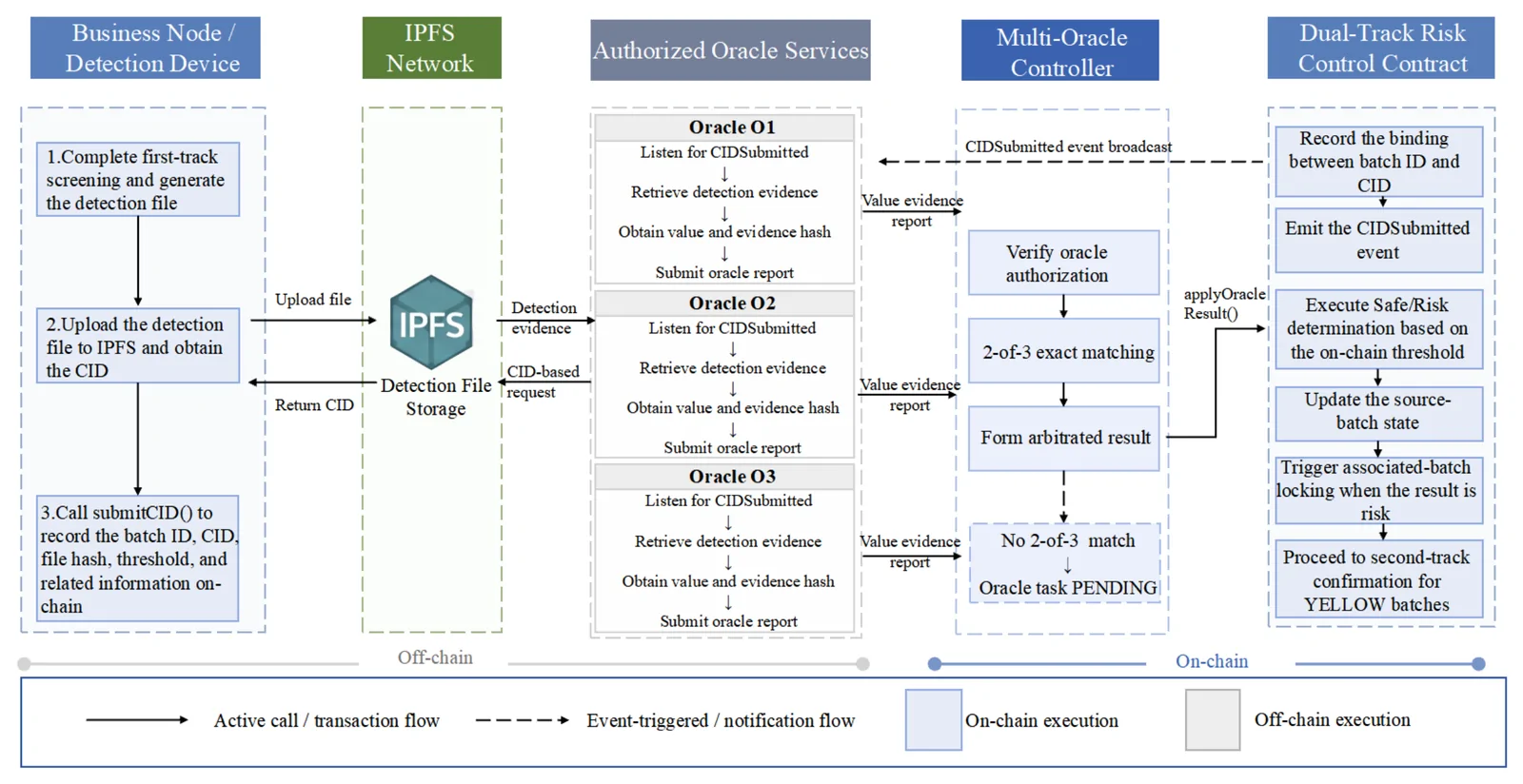
InterPlanetary File System (IPFS)-based evidence retrieval and two-of-three multi-oracle result arbitration.

The above process separates evidence retrieval from on-chain state determination: individual oracle reports cannot directly update the batch state, and the subsequent threshold rule is executed only after the two-of-three value–evidence matching condition is satisfied. The complete interaction among IPFS, the authorized oracle services, the multi-oracle controller, and the dual-track contract is shown in Figure 3.

In summary, the mechanism combines IPFS-based off-chain storage, CID anchoring, and two-of-three evidence-bound oracle arbitration before an externally supplied result can affect batch state. This reduces dependence on a single oracle while retaining the trust boundary that blockchain does not independently verify the analytical correctness of the upstream measurement.

### 2.3. Hash-Based Inverted-Index Mechanism for Associated-Batch Locking

In food supply-chain risk control, the impact scope of a problematic batch depends not only on its detection result but also on the processing equipment, storage locations, transport vehicles, and operational units used during circulation. Blocking only the problematic batch may overlook potentially risky batches sharing equipment, whereas full-batch, same-node, or historical same-device locking may cause over-blocking. This study therefore designs a hash-based inverted-index mechanism that identifies batches with explicit shared-equipment relationships after a risk source is detected and updates them to the YELLOW pending-confirmation state.

The mechanism converts batch device-usage records from different supply-chain stages into hash index keys and establishes an inverted mapping between index keys and batch sets. When the smart contract classifies a batch as RED based on an arbitrated oracle result, or the second track confirms it as risky, the system queries the associated-batch set through the corresponding index keys. Matched batches are not directly classified as abnormal; instead, they undergo second-track re-examination and are subsequently restored to GREEN or updated to RED according to the confirmation result.

Let the food batch set be defined as

(14) $$B=\{b_{1},b_{2},\cdots ,b_{n}\}$$

Let the supply chain stage set be defined as

(15) $$S=\{s_{1},s_{2},\cdots ,s_{m}\}$$

and let the device set be defined as

(16) $$D=\{d_{1},d_{2},\cdots ,d_{k}\}$$

When batch $b_{i}$ uses device $d_{k}$ at stage $s_{j}$, the system generates a device usage record:

(17) $$r_{i}=\left(b_{i},s_{j},d_{k},p,t\right)$$

where $b_{i}$ denotes the food batch identifier, $s_{j}$ denotes the supply chain stage identifier, $d_{k}$ denotes the device identifier, p denotes the operation identifier, and t denotes the recording time. It should be noted that p in this study does not represent a time slice. Instead, it refers to an operation order, processing task, storage task, or transportation task identifier that can be recorded in the supply chain business system. The purpose of introducing the operation identifier is to avoid including all batches processed by the same device over its entire historical period in the locking scope, thereby reducing the risk of over-locking.

For each device usage record, the system generates a hash index key according to the stage identifier, device identifier, and operation identifier:

(18) $$K_{j,k,p}=H(s_{j}∥d_{k}∥p)$$

where H(⋅) denotes the hash function, and ∥ denotes string concatenation. This index key is used to represent the shared-equipment relationship under “a specific supply chain stage, a specific device, and a specific operation task”. Compared with an indexing method that uses only the device identifier, this design additionally incorporates the stage and operation identifiers, enabling the system to distinguish the usage relationships of the same device across different stages and different operation tasks.

The system then constructs the inverted index as follows:

(19) $$I(K)=\{b|K\in T(b_{i})\}$$

where $T(b_{i})$ denotes the set of index keys generated by batch $b_{i}$ during supply chain circulation, and I(K) denotes the batch set corresponding to index key K. In other words, all batches that appear in the same stage, use the same device, and belong to the same operation task are mapped to the same index key.

For any batch $b_{i}$, its device index key set can be expressed as

(20) $$T(b_{i})=\{K_{j,k,p}|b_{i}\;\mathrm{uses}\;d_{k}\;\mathrm{at}\;s_{j}\;\mathrm{under}\;\mathrm{task}\;p\}$$

When the smart contract determines batch $b_{x}$ as RED based on the arbitrated oracle value, or when the second-track confirmation mechanism confirms batch $b_{x}$ as abnormal, the system regards $b_{x}$ as the risk-source batch. At this point, the system first reads the index key set $T(b_{x})$ of this batch, and then performs an inverted query for each index key in the set to obtain the associated-batch set that needs to be temporarily locked:

(21) $$B_{\mathrm{lock}}=\left(\underset{K\in T(b_{x})}{\cup }I(K)\right)-\{b_{x}\}$$

where $B_{\mathrm{lock}}$ denotes the set of batches that have shared-equipment relationships with the problematic batch $b_{x}$. The batch $b_{x}$ itself is removed from this set because the risk-source batch has already been updated to RED and does not need to enter the associated-batch locking process again.

For the batches in $B_{\mathrm{lock}}$, the system does not directly determine them as abnormal. Instead, it updates them to the YELLOW state:

(22) $$\forall b_{i}\in B_{\mathrm{lock}},\;\;\;\;\mathrm{State}(b_{i})=\mathrm{YELLOW}$$

The YELLOW state indicates that the batch has an associated risk due to shared equipment, but has not yet been confirmed as unqualified. Therefore, the system only suspends its further circulation and requires it to enter the second-track rapid confirmation process. Only when the second-track confirmation data are legal and the result is qualified can the batch be restored to GREEN. If the confirmation result is unqualified, the batch is updated to RED. In this way, the hash-based inverted index completes the directed mapping from the risk-source batch to the associated-batch set, and all matched batches are uniformly placed into the YELLOW pending-confirmation state.

Full-batch, same-node, and historical same-device locking respectively include all non-source batches, all batches within the same supply-chain node, or all batches that previously used the same device in the risk-control scope. In contrast, the proposed method locks only batches associated with the risk source under the same stage, device, and operation identifier, thereby reducing over-blocking while maintaining potential-risk coverage.

**Figure 4:**
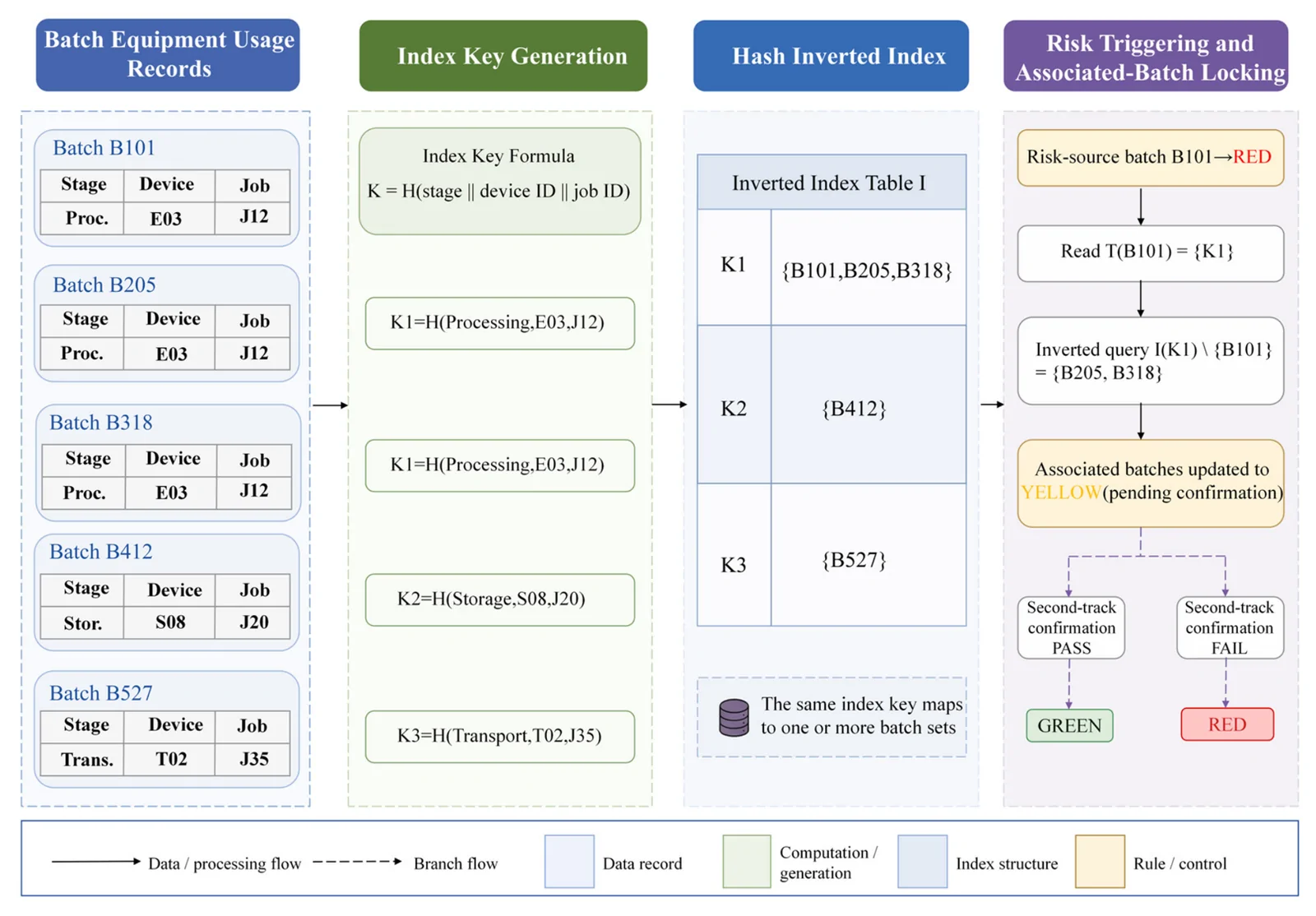
Schematic diagram of the hash-based inverted-index mechanism for associated-batch locking.

Figure 4 illustrates associated-batch locking using a scenario in which batches share processing, storage, or transportation equipment. Batches B101, B205, and B318 use device E03 at the same processing stage under operation identifier J12 and are therefore mapped to index key K1; B412 and B527 are mapped to other keys because their stage, device, or operation identifier differs. After B101 is identified as a risk source by the left or second track and updated to RED, the smart contract queries the batch set corresponding to K1. After excluding B101, the contract identifies B205 and B318 as associated batches and updates them to YELLOW. The locking scope is thus limited to batches sharing the same stage, device, and operation identifier, avoiding unnecessary blocking caused by coarse-grained strategies.

### 2.4. Second-Track Signature-Based Confirmation Mechanism

For YELLOW batches matched by the hash-based inverted index, the system must determine whether the associated risk is confirmed before circulation is restored or blocked. Directly classifying all associated batches as abnormal would cause over-blocking, whereas restoring circulation based on unverified confirmatory results would create a risk of forged release. This study therefore designs a second-track signature-based confirmation mechanism that uses device signatures to ensure source authenticity and content integrity. Unlike the high-dimensional screening files used in the left track, second-track data are small-scale structured records containing the batch identifier, hazard type, detection value, threshold, device identifier, timestamp, and conclusion, and can therefore be submitted directly to the smart contract for signature verification, authorization checking, and state updating.

Let the second-track confirmatory data be denoted as $C_{i}$, whose field set can be expressed as

(23) $$C_{i}=(b_{i},h_{i},v_{i},\theta_{i},d_{i},\tau_{i},y_{i})$$

where $b_{i}$ denotes the batch identifier, $h_{i}$ denotes the hazard type, $v_{i}$ denotes the detection value, $\theta_{i}$ denotes the determination threshold, $d_{i}$ denotes the confirmatory device identifier, $\tau_{i}$ denotes the timestamp, and $y_{i}$ denotes the confirmation conclusion, with

(24) $$y_{i}\in \{\mathrm{PASS},\mathrm{FAIL}\}$$

After generating the structured result, the confirmatory device first computes the data digest:

(25) $$M_{i}=H(b_{i}∥h_{i}∥v_{i}∥\theta_{i}∥d_{i}∥\tau_{i}∥y_{i})$$

where H(⋅) denotes the hash function, ∥ denotes field concatenation, and $M_{i}$ denotes the digest of the confirmatory data. The device then signs the digest using its private key:

(26) $$\sigma_{i}={\mathrm{Sign}}_{sk_{d_{i}}}(M_{i})$$

where $sk_{d_{i}}$ denotes the private key corresponding to device $d_{i}$, and $\sigma_{i}$ denotes the device signature. After receiving the confirmatory data, the smart contract verifies the signature using the authorized device public key or the corresponding on-chain address:

(27) $$\mathrm{Verify}(pk_{d_{i}},M_{i},\sigma_{i})=\mathrm{true}$$

where $pk_{d_{i}}$ denotes the public key corresponding to device $d_{i}$. If any field in the submitted data is modified after signing, the recomputed data digest will not match the signature, causing the contract-side signature verification to fail. Consequently, the confirmatory result is rejected.

In addition to signature verification, the smart contract must also verify whether the submitting device belongs to the authorized device set. Let $D_{\mathrm{auth}}$ denote the authorized device set. The device authorization condition is therefore defined as

(28) $$d_{i}\in D_{\mathrm{auth}}$$

Meanwhile, the contract needs to check whether the detection value is consistent with the confirmation conclusion. In this study, the result consistency condition is defined as

(29) $$\mathrm{Consistent}(C_{i})=\left\{\begin{array}{cc} \mathrm{true}, & v_{i}\leq \theta_{i}\wedge y_{i}=\mathrm{PASS}\\ \mathrm{true}, & v_{i}>\theta_{i}\wedge y_{i}=\mathrm{FAIL}\\ \mathrm{false}, & \mathrm{otherwise} \end{array}\right.$$

In addition, to prevent repeated confirmation of the same batch, the system maintains a confirmation status flag for each YELLOW batch. Let $\mathrm{Confirmed}(b_{i})$ indicate whether batch $b_{i}$ has already completed confirmation. A legal submission must further satisfy

(30) $$\mathrm{Confirmed}(b_{i})=\mathrm{false}$$

Based on the above rules, the contract-side verification process for second-track confirmation includes four types of checks: whether the signature matches, whether the device is authorized, whether the detection value is consistent with the result, and whether the batch has been repeatedly submitted. Only after all checks are passed does the contract update the batch state according to the confirmation conclusion. The corresponding verification rules are summarized in Table 3.

After the verification process is completed, the contract updates the batch state according to the relationship between the detection value and the threshold. If the detection value does not exceed the threshold and the result is PASS, the batch is restored from YELLOW to GREEN. If the detection value exceeds the threshold and the result is FAIL, the batch is updated from YELLOW to RED. The state update rule can be expressed as

(31) $$\mathrm{State}(b_{i})=\left\{\begin{array}{cc} \mathrm{GREEN}, & \mathrm{State}(b_{i})=\mathrm{YELLOW}\wedge v_{i}\leq \theta_{i}\wedge y_{i}=\mathrm{PASS}\wedge \mathrm{Valid}(C_{i})\\ \mathrm{RED}, & \mathrm{State}(b_{i})=\mathrm{YELLOW}\wedge v_{i}>\theta_{i}\wedge y_{i}=\mathrm{FAIL}\wedge \mathrm{Valid}(C_{i})\\ \mathrm{YELLOW}, & \mathrm{State}(b_{i})=\mathrm{YELLOW}\wedge \neg \mathrm{Valid}(C_{i}) \end{array}\right.$$

where $\mathrm{Valid}(C_{i})$ indicates that the confirmatory data simultaneously satisfy the requirements of signature integrity, device authorization, result consistency, and non-duplicate submission. It is defined as

(32) $$\mathrm{Valid}(C_{i})=\mathrm{Verify}(pk_{d_{i}},M_{i},\sigma_{i})\wedge (d_{i}\in D_{\mathrm{auth}})\wedge \mathrm{Consistent}(C_{i})\wedge (\mathrm{Confirmed}(b_{i})=\mathrm{false})$$

These rules prevent YELLOW batches from being released on the basis of unverified results or being classified as abnormal solely because they were matched by the index. The second track therefore provides authenticated rule enforcement: LEGAL_PASS records restore the batch to GREEN, LEGAL_FAIL records update it to RED, and ILLEGAL or inconsistent submissions are rejected while the batch remains YELLOW.

In summary, the dual-track mechanism comprises high-dimensional file access, multi-oracle result arbitration, contract-based state determination, associated-batch locking, and second-track signature confirmation. The complete workflow is summarized in Algorithm 1.
**Algorithm 1.** Overall workflow of the dual-track risk-control mechanism$\mathrm{Input}:\;\mathrm{batchId},\;\mathrm{detection}\;\mathrm{file}\;F_{i}$, CID, valueScaled, oracle reports, equipmentIndexKeys, confirmationData Output: final batch state 1. Upload $F_{i}$ to IPFS, obtain the CID, and bind the CID to batchId on-chain. 2. Emit CIDSubmitted, and let the three authorized oracles independently retrieve the CID-addressed evidence. 3. Each oracle submits valueScaled and evidenceHash using its authorized identity. 4. Verify oracle authorization and apply the result only when at least two reports contain an exactly matching (valueScaled, evidenceHash) pair. 5. If no 2-of-3 match is formed, keep the oracle task PENDING without applying a new batch state. 6. Compare the arbitrated valueScaled with thresholdScaled. 7. If the result satisfies the normal condition, set or maintain the source batch as GREEN. 8. If the result satisfies the risk condition, update the source batch to RED and query associated batches through equipmentIndexKeys. 9. Update the matched associated batches to YELLOW. 10. For each YELLOW batch, verify the confirmation signature, device authorization, result consistency, state precondition, and duplicate-submission status. 11. If the confirmation is LEGAL_PASS, update YELLOW → GREEN. 12. If the confirmation is LEGAL_FAIL, update YELLOW → RED; if the submission is ILLEGAL, reject it and retain YELLOW. 13. Return the final batch state.

## 3. Experimental Design

### 3.1. Experimental Environment and Workflow Configuration

To validate the proposed trusted-circulation and dual-track risk-control mechanism, an Ethereum-compatible experimental environment is constructed around the workflow of “high-dimensional detection data access—multi-oracle result arbitration—associated-batch locking—second-track signature-based confirmation—on-chain state updating.” The experiments evaluate CID notarization, multi-oracle arbitration, hash-based inverted indexing, confirmation-signature verification, and batch-state updating.

The system experiments use a four-node Hyperledger Besu 25.11.0 QBFT permissioned blockchain, three separate Kubo/IPFS repositories, and three logical oracle services as the on-chain/off-chain execution environment. The .bil/.hdr files and contamination concentration labels from the HyperPistachio dataset [33] serve as real left-track inputs. Each oracle uses an independently authorized identity to retrieve CID-addressed files and submit the detection value together with its evidence hash; a result enters subsequent state control only when at least two of the three oracles provide exactly matching valueScaled and evidenceHash. Hardhat 2.28.6 is used for contract compilation, unit testing, state-machine semantic validation, and controlled workload characterization, including latency, throughput, and queuing behavior under normal- and high-load settings. The multi-node, multi-oracle, fault-injection, real-file-driven, confirmation-order, and sustained-operation experiments are conducted on the four-node Besu-QBFT network. The experimental environment and tool configuration are summarized in Table 4.

The smart-contract layer comprises a multi-oracle controller and the dual-track risk-control contract. The former manages oracle authorization, result submission, 2-of-3 arbitration, and evidence-hash binding; the latter performs batch registration, CID notarization, device-index maintenance, associated-batch locking, second-track confirmation, and GREEN/YELLOW/RED state updating. Its main functions are listed in Table 5.

The experimental workflow comprises three stages: left-track file access, associated-batch locking, and second-track confirmation. The edge node first uploads a high-dimensional detection file to IPFS and submits its CID. The authorized oracles retrieve the CID-addressed file and independently submit their results, after which the 2-of-3 controller forms an arbitrated result for threshold determination and state updating. Once a risk-source batch is updated to RED, the system queries associated batches using the composite index of “supply-chain stage + device identifier + operation identifier” and locks them in YELLOW. A confirmatory device then submits structured results and a signature, and the contract verifies signature integrity, device authorization, result consistency, and duplicate-submission status before restoring the batch to GREEN, updating it to RED, or retaining it in YELLOW. All Besu-QBFT multi-node experiments are conducted using a controlled single-host logical multi-node deployment; therefore, these results characterize logical multi-node prototype execution rather than cross-institutional wide-area-network deployment.

### 3.2. Data Sources, AFB1 Labels, and Experimental Boundaries

The experiments use real HyperPistachio hyperspectral files and concentration labels together with controlled supply-chain association and second-track confirmation data. The real files provide the input basis for evaluating high-dimensional detection-file access and on-chain/off-chain workflow execution, whereas the controlled data are used only to configure device–operation relationships and confirmation scenarios.

This study evaluates trusted access to detection data, multi-oracle result formation, contract-based threshold determination, on-chain state updating, and the risk-control loop rather than a food hazard recognition model. This study uses 57 independent HyperPistachio samples with explicit AFB1 concentration labels. Each sample contains one band-interleaved-by-line (BIL) hyperspectral file, one header (HDR) metadata file, and one Portable Network Graphics (PNG) preview image, giving 57 BIL, 57 HDR, and 57 PNG files. Each BIL file is 90,832,896 B equivalent to 86.625 mebibytes (MiB), and the 57 BIL files total approximately 4.82 gibibytes (GiB). The BIL/HDR files serve as real left-track inputs for validating off-chain storage, CID-based retrieval, oracle processing, and source-batch threshold determination. Controlled stage–device–operation relationships and structured confirmation records are subsequently used to exercise associated-batch locking and second-track state control. The AFB1 concentration values are taken directly from the original dataset; this study neither re-measures the concentrations nor trains or evaluates a new hyperspectral AFB1 detection or quantification model. Table 6 and Table 7 summarize the data sources, experimental purposes, and interpretation boundaries.

Accordingly, the experimental validation in this study is limited to AFB1-related detection data from pistachio samples in the HyperPistachio dataset. The results are not extended to pesticide residues, veterinary drug residues, heavy metals, allergens, or other food hazards without further validation using corresponding detection data and business rules. Under the 10.0 μg/kg system screening threshold used for workflow validation, the 57 independent samples comprise 51 GREEN and 6 RED inputs. GREEN indicates only that the supplied AFB1 value does not trigger the RED state under the predefined experimental rule; it does not certify the absence of other contaminants or the overall safety of the food.

Repeated workflow execution is used only to evaluate system-level behavior and does not increase the number of independent food samples. For end-to-end dual-track evaluation, the same 57 independent inputs are reused across five system-level rounds, producing 285 workflow executions. These executions are used to examine source-batch determination, associated-batch locking, second-track confirmation, and the effect of confirmation order on the final state. In the sustained-operation experiment, the same 57 independent inputs are further executed across 10 rounds, producing 570 workflows for evaluating repeated fixed-contract execution, resource behavior, and multi-node state consistency. Neither workflow count represents additional independent biological observations.

### 3.3. Experimental Modules and Evaluation Framework

The proposed framework is evaluated in terms of real-file IPFS-CID access and oracle processing, hash-based inverted-index locking, second-track LEGAL/ILLEGAL confirmation, end-to-end multi-node execution, sustained operation, and a HyperPistachio real-file-driven case. The experimental modules, objectives, and evaluation metrics are summarized in Table 8.

The experiments follow the sequence of “real detection-file access—trusted oracle result formation—associated-batch locking—second-track confirmation—multi-node state updating—real-file-driven case validation.” In Section 4.2, real HyperPistachio files are used to evaluate IPFS-CID access and storage boundaries, while single-oracle and three-oracle 2-of-3 modes are further compared under normal and fault conditions. Section 4.3 compares full-batch, same-node, historical same-device, and stage–device–operation locking strategies and evaluates associated-batch fanout and the corresponding execution overhead. Section 4.4 evaluates second-track confirmation using LEGAL_PASS, LEGAL_FAIL, and ILLEGAL submissions, with ILLEGAL cases covering tampering, unauthorized devices, inconsistent results, and duplicate submissions. Section 4.5 first characterizes end-to-end workflow latency, throughput, and queuing behavior under controlled Hardhat-based workload conditions. It then evaluates real-file-driven dual-track execution, confirmation-order behavior, replicated-state consistency, and sustained fixed-contract operation in the four-node Besu-QBFT environment. Finally, Section 4.6 returns to the 57 independent HyperPistachio samples to interpret the real-file and AFB1-data basis of the case, the resulting input-state distribution, and the corresponding evidence boundaries.

The meanings of the evaluation metrics are strictly separated. Locking reduction and fanout metrics characterize associated-batch control; LEGAL/ILLEGAL outcomes characterize enforcement of second-track contract rules; GREEN/YELLOW/RED states characterize execution of the predefined state machine.

All Besu-QBFT multi-node experiments are conducted in the controlled single-host logical multi-node environment described in Section 3.1. Accordingly, these results characterize prototype executability, fault tolerance, tested-range scalability, and sustained operation rather than cross-institutional wide-area network (WAN) performance or production-scale deployment.

## 4. Results and Analysis

### 4.1. Overall Correspondence of Experimental Results

Section 4 follows the sequence of trusted off-chain file access and multi-oracle arbitration, associated-batch locking, second-track confirmation, end-to-end validation, and real-file case validation. Section 4.2 evaluates IPFS-CID storage and access using real high-dimensional files and further examines the performance and fault boundary of single- and multi-oracle modes. Section 4.3 evaluates the locking scope of the hash-based inverted index and its state-control and fanout characteristics. Section 4.4 evaluates second-track signature-based confirmation using LEGAL and ILLEGAL submissions. Section 4.5 first characterizes end-to-end workflow behavior under controlled Hardhat-based normal- and high-load conditions and then evaluates real-file-driven dual-track execution, second-track confirmation-order behavior, Besu-QBFT multi-node state consistency, and sustained operation. Section 4.6 interprets the 57 independent HyperPistachio inputs as the food-data basis of the AFB1 case and relates these independent inputs to the system-level workflow evidence. Section 4.7 compares the proposed mechanism with conventional digital management approaches and discusses its practical deployment boundary.

### 4.2. Performance Analysis of IPFS-CID Notarization, Real-File Access, and Oracle Processing

**Figure 5:**
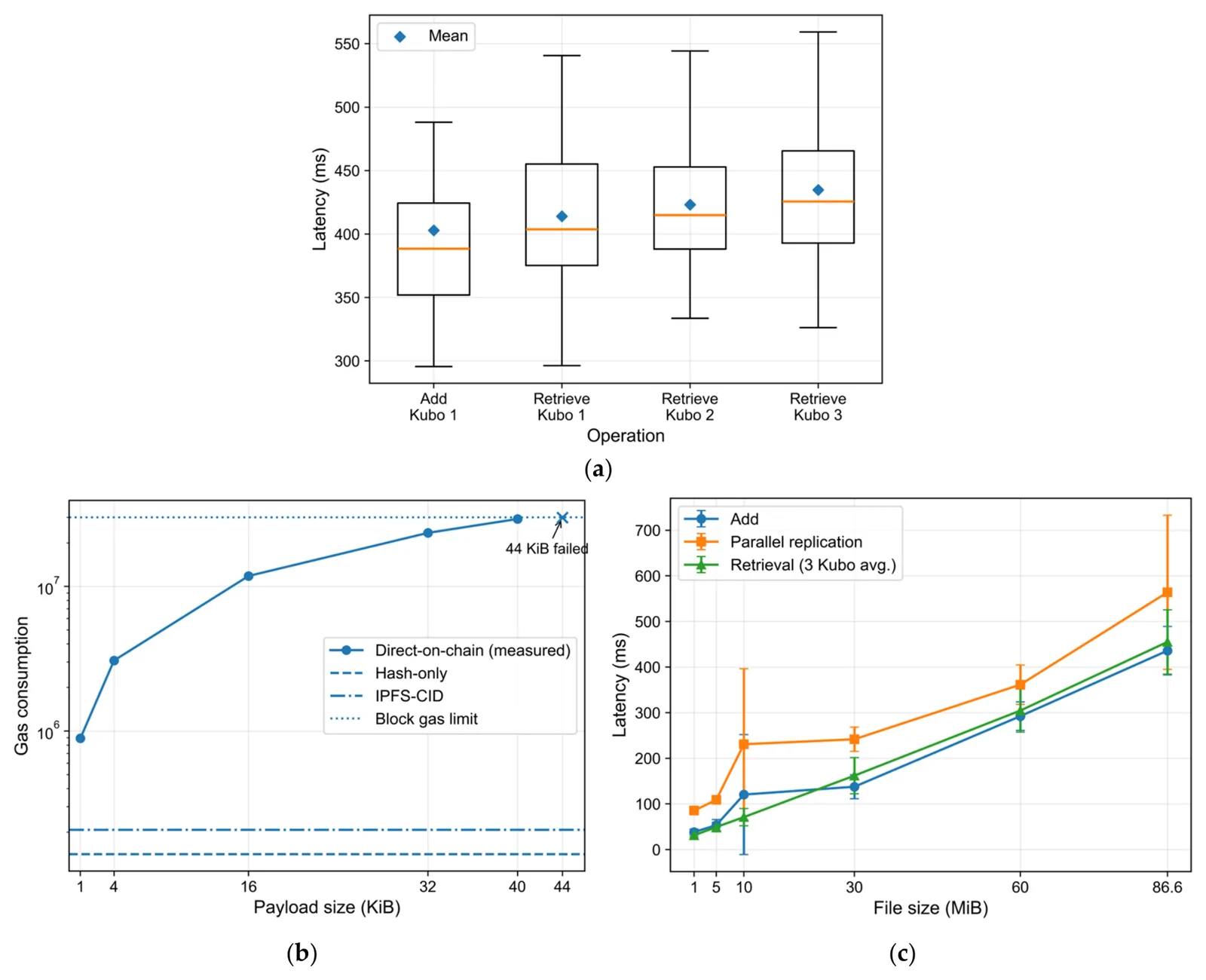
Real aflatoxin B1 (AFB1) hyperspectral-file access and on-chain storage boundary: (**a**) InterPlanetary File System (IPFS) add and retrieval latency of 57 real band-interleaved-by-line (BIL) files across three Kubo repositories; (**b**) Direct-on-chain Gas consumption and transaction-capacity boundary; (**c**) IPFS processing latency under different file sizes using unique objects.

**Figure 6:**
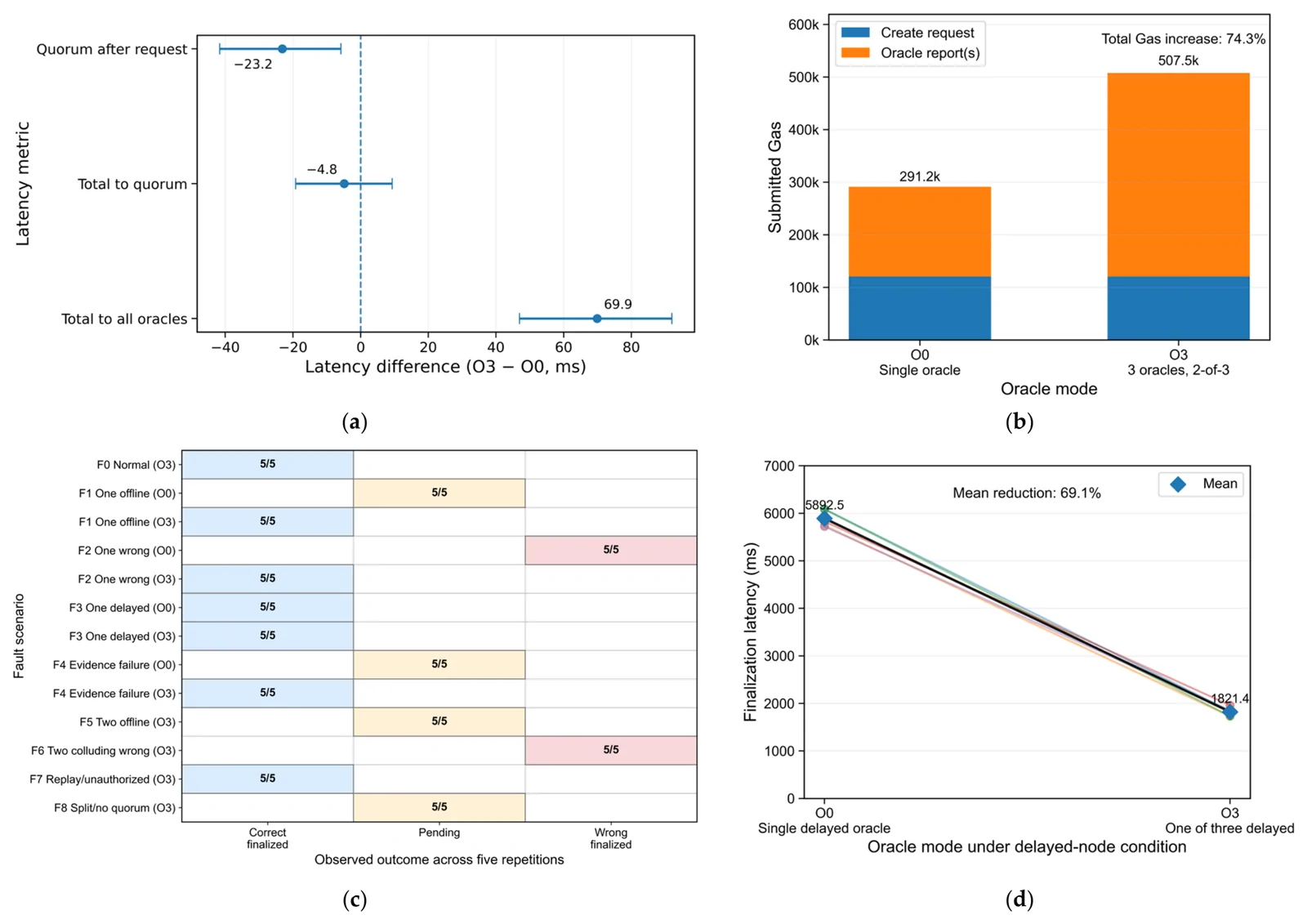
Performance and fault behavior of single- and multi-oracle modes: (**a**) paired latency differences between the three-oracle mode (O3) and the single-oracle mode (O0) under normal conditions; (**b**) submitted Gas consumption for request creation and oracle reporting in O0 and O3; (**c**) observed outcomes under the tested oracle fault scenarios across five repetitions; (**d**) finalization latency under the delayed-node condition for O0 with its single oracle delayed and O3 with one of three oracles delayed. In (**d**), the thin colored lines represent the five paired experimental repetitions, the black line connects the mean finalization latencies, and the blue diamonds denote the mean values.

This section evaluates the role of IPFS-CID in incorporating high-dimensional detection files into the on-chain state-control process and examines the performance and trust boundary of oracle processing. The evaluation compares Direct-on-chain, Hash-only, and IPFS-CID storage modes and examines both real HyperPistachio file processing and three-oracle two-of-three arbitration. The results are presented in Figure 5 and Figure 6 and Table 9 and Table 10.

Figure 5a evaluates 57 real BIL files across three separate Kubo repositories. Each BIL file is 90,832,896 B (86.625 MiB). The mean add time in Kubo 1 is 402.916 ms, while the mean retrieval times from Kubo 1, Kubo 2, and Kubo 3 are 414.038, 423.185, and 434.753 ms, respectively. File-size and hash verification were successful for all files, indicating that the real hyperspectral files could be consistently stored and retrieved in the controlled multi-Kubo environment. Because the three repositories run on the same physical host, these values characterize prototype file access rather than cross-institutional network-transfer latency.

The comparison among the three storage modes supports the storage design choice. Direct-on-chain stores file contents directly in the blockchain state, whereas Hash-only and IPFS-CID retain only compact metadata on-chain. As shown in Figure 5b, Direct-on-chain transactions succeed for payloads up to 40 kibibytes (KiB), consuming 29,245,745 Gas at 40 KiB, but fail at 44 KiB under the 30,000,000 block Gas limit. By contrast, the mean Gas consumption of Hash-only and IPFS-CID is approximately 140.7 thousand and 207.9 thousand, respectively. Extrapolation from the successful Direct-on-chain measurements gives an estimated Gas requirement of approximately 6.45 × 10^10^ for a 90.83 MB BIL file; this value is a projection rather than an executed large-file transaction. Thus, the principal advantage of IPFS-CID is not lower Gas than Hash-only, but the ability to retain a content-addressed retrieval entry while decoupling the on-chain data volume from the original file size.

Figure 5c further evaluates IPFS processing with unique SHAKE-256 objects to reduce the influence of content deduplication. As file size increases from 1 to 86.625 MiB, the mean add time increases from 38.264 to 435.955 ms, and the mean retrieval time across the three Kubo repositories increases from 31.178 to 455.108 ms; parallel replication of the 86.625 MiB objects requires 563.928 ms on average. These results indicate that the additional cost of real high-dimensional files is concentrated mainly in off-chain storage, replication, and retrieval rather than in CID anchoring itself.

After establishing the real-file access boundary, the oracle experiments evaluate whether multi-oracle arbitration can reduce dependence on a single authorized oracle. O0 uses one authorized oracle, whereas O3 uses three independently authorized logical oracles and forms an accepted result when at least two provide exactly matching valueScaled and evidenceHash. Both modes are evaluated using the same 57 real HyperPistachio file inputs over five paired rounds.

Under normal operating conditions, the mean total times from request creation through quorum finalization are 3782.88 ms for O0 and 3778.03 ms for O3, respectively. The paired O3–O0 difference is −4.84 ms, with a 95% bootstrap confidence interval of [−19.21, 9.32] ms. The interval crosses zero, indicating no clear increase in final arbitration latency for O3 under the tested conditions. However, multiple oracle reports increase the on-chain execution cost. As shown in Figure 6b, task-creation Gas remains similar between the two modes, whereas the reporting stage increases because O3 requires multiple authorized submissions. The mean total submitted Gas therefore increases from approximately 291.20 thousand in O0 to 507.53 thousand in O3, corresponding to an increase of approximately 74.3%. Multi-oracle arbitration consequently trades additional on-chain reporting cost for reduced dependence on a single authorized oracle service.

Figure 6c summarizes the oracle fault experiments. A single offline oracle, a single incorrect oracle, a delayed oracle, or a single evidence-validation failure does not prevent O3 from reaching the expected result when two consistent reports remain available. In contrast, O0 remains PENDING when its sole oracle is offline or fails evidence validation, while an incorrect result from the sole oracle produces an incorrect final state in all five repetitions.

The two-of-three mechanism also has a clear failure boundary. When two O3 oracles are simultaneously offline, or when the submitted results are split and no two identical reports are available, the task remains PENDING. When two authorized oracles collude and submit the same incorrect result, an incorrect final state is formed in all five repetitions. Unauthorized replay does not alter the expected state. Across all tested fault conditions, the four Besu nodes maintain mutually consistent on-chain states, showing that oracle failure affects the validity or availability of the input result rather than the consistency of the replicated blockchain state.

The effect of oracle delay is shown in Figure 6d. When the sole O0 oracle is delayed, the mean finalization time increases to 5892.55 ms. In O3, when only one of the three oracles is delayed, the other two can satisfy the two-of-three arbitration condition, and the mean finalization time is 1821.44 ms, approximately 69.1% lower than that of the delayed O0 condition. This result does not indicate faster oracle computation; instead, it shows that final state formation no longer depends on the response of a single delayed oracle.

Overall, the real-file experiments show that the main cost of processing 86.625 MiB hyperspectral files lies in off-chain storage, replication, and retrieval, while CID anchoring keeps the on-chain data volume independent of the original file size. The oracle experiments further show that two-of-three arbitration can tolerate a single unavailable, incorrect, delayed, or evidence-failing oracle, but it does not eliminate the trust risk arising from a majority of authorized oracles providing the same incorrect result. Therefore, the multi-oracle mechanism reduces the single-point trust dependency of oracle-based state control rather than providing an absolute guarantee of the correctness of off-chain detection results.

### 4.3. Analysis of Locking Scope and Scalability of the Hash-Based Inverted Index

**Figure 7:**
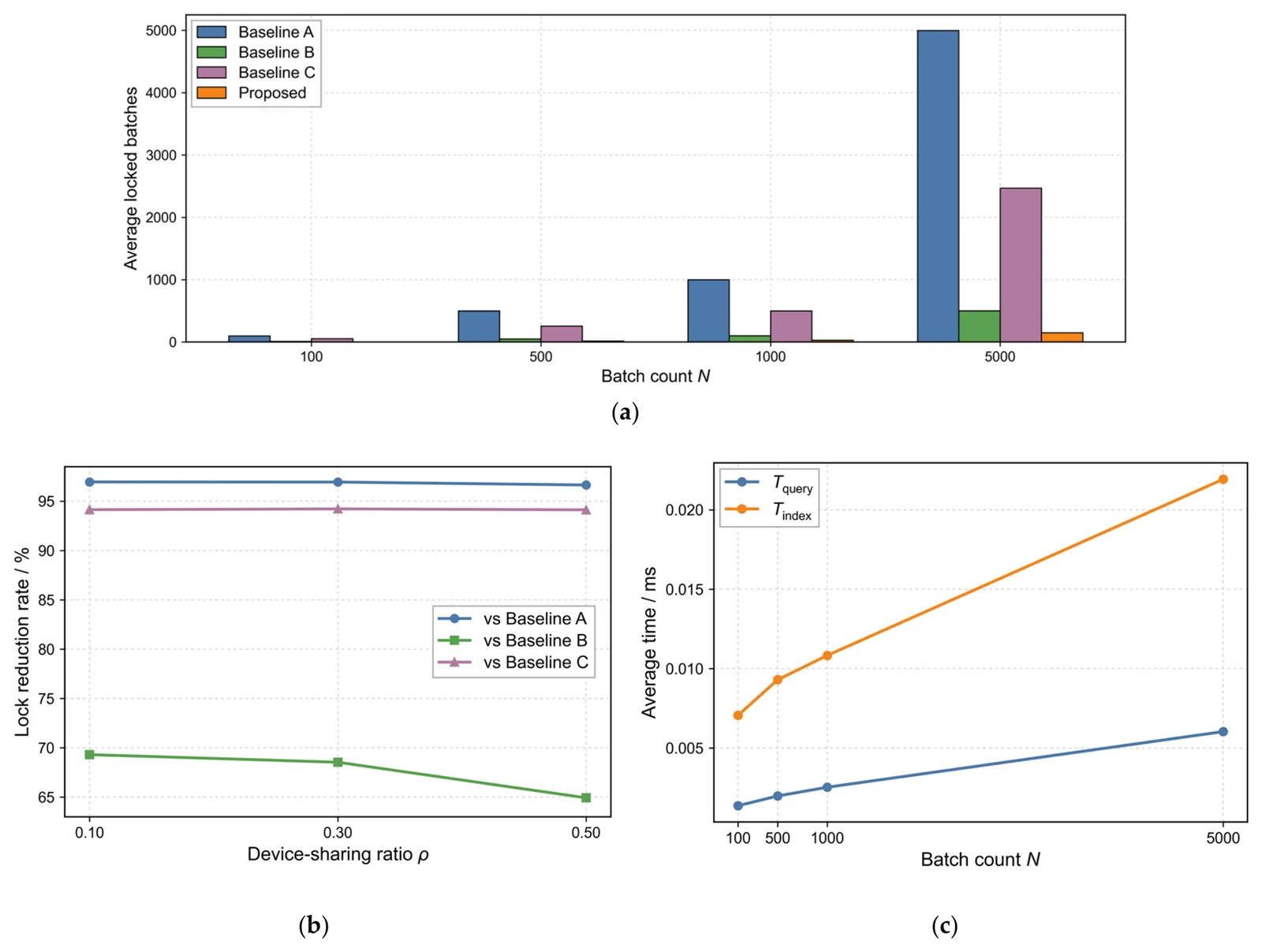
Locking-scope comparison and index-query behavior of the hash-based inverted index: (**a**) comparison of the number of locked batches between Baselines A/B/C and Proposed; (**b**) locking reduction rate of Proposed relative to the three baselines; (**c**) index-query time under different data scales.

The hash-based inverted index is used to selectively identify associated batches defined by the recorded stage–device–operation relationship. To characterize its locking scope and execution behavior, four risk-locking strategies are compared: Baseline A represents full-batch locking, Baseline B represents same-node locking, Baseline C represents historical same-device locking, and the Proposed strategy represents hash-based inverted-index locking based on “stage + device + operation identifier”. The number of constructed batches is set as N∈{100,500,1000,5000}, the number of supply-chain stages is set to six, the number of devices per stage is set as D∈{5,10,20}, the number of operation identifiers is set as P∈{10,50,100}, and the device-sharing ratio is set as ρ∈{0.10,0.30,0.50}. In each experimental round, one batch is randomly selected as the risk-source batch, and the controlled reference associated set is defined according to its “stage + device + operation identifier” relationship. This set is then used to compare the locking scope of the four strategies. The experimental results are shown in Figure 7 and Table 11 and Table 12.

Figure 7a and Table 11 show that the locking scopes of all three baseline strategies increase markedly with batch scale. At N = 100, the mean numbers of batches locked by Baselines A, B, C, and Proposed are 99.00, 9.89, 54.10, and 3.45, respectively; at N = 5000, they increase to 4999.00, 501.22, 2467.81, and 148.07. Baseline A locks almost all non-source batches, Baseline C includes many batches that historically used the same device, and Baseline B remains a coarse-grained node-level strategy despite its smaller scope. Proposed consistently produces the smallest locking set and more closely follows the predefined shared-equipment relationships in the controlled topology.

Figure 7b and Table 12 show that, at device-sharing ratios of ρ = 0.10, 0.30, and 0.50, the locking reductions of Proposed relative to Baseline A are 96.96%, 96.95%, and 96.65%, respectively. The corresponding reductions relative to Baseline B are 69.31%, 68.54%, and 64.93%, while those relative to Baseline C remain above 94%. Under the controlled experimental topology, all batches defined by the same stage–device–operation relationship are consistently retrieved without additional matches. This result reflects deterministic agreement with the predefined association rule rather than empirical accuracy in identifying contamination relationships in real supply chains.

Figure 7c shows that, as the number of batches increases from 100 to 5000, total index processing time rises only from 0.007063 ms to 0.021932 ms, while inverted-query time increases from 0.001371 ms to 0.006036 ms. By locating associated-batch sets directly through index keys, the hash-based inverted index avoids full traversal and maintains low query overhead as the batch scale increases.

**Figure 8:**
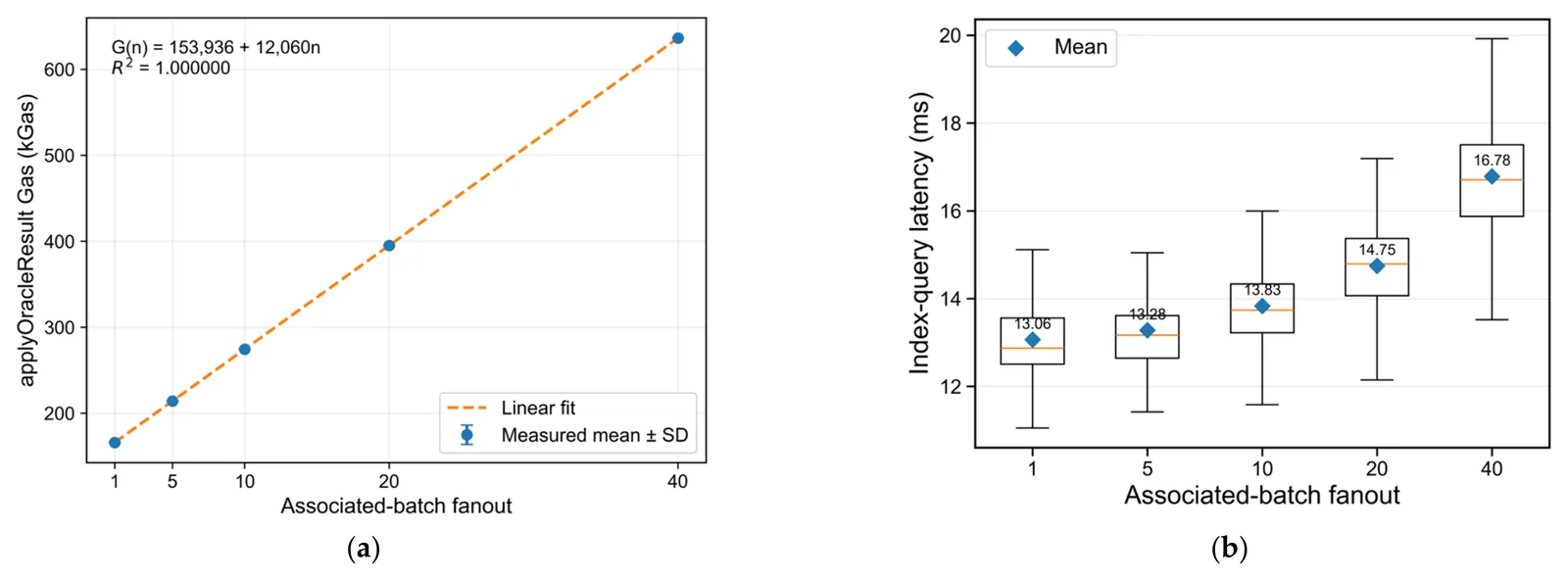
Scalability of associated-batch propagation under different fanout levels: (**a**) Gas consumption under different associated-batch fanout levels; (**b**) index-query latency under different associated-batch fanout levels.

To further examine scalability with respect to the number of associated batches, the fanout of a single risk source was varied from 1 to 40. As shown in Figure 8a, the mean Gas consumption of applyOracleResult() increased from 165,993 at fanout = 1 to 636,345 at fanout = 40, showing an approximately linear increase in on-chain state-update cost within the tested range. In contrast, Figure 8b shows that the mean index-query latency increased only from 13.06 to 16.78 ms as fanout increased from 1 to 40. These results indicate that increasing the number of associated batches mainly affects the cost of on-chain state updates, whereas the additional index-query overhead remains comparatively limited. The tested fanout range is used to characterize prototype scalability and is not interpreted as a production-scale performance limit.

Overall, the proposed index reduced the locking scope relative to all three baseline strategies, while increasing fanout mainly raised on-chain state-update cost and produced only a limited increase in query latency within the tested range. Agreement with the controlled stage–device–operation topology reflects deterministic execution of the predefined association rule rather than empirical accuracy in identifying contamination relationships in real supply chains.

### 4.4. Analysis of Second-Track Signature-Based Confirmation and Tamper-Resistance Results

**Figure 9:**
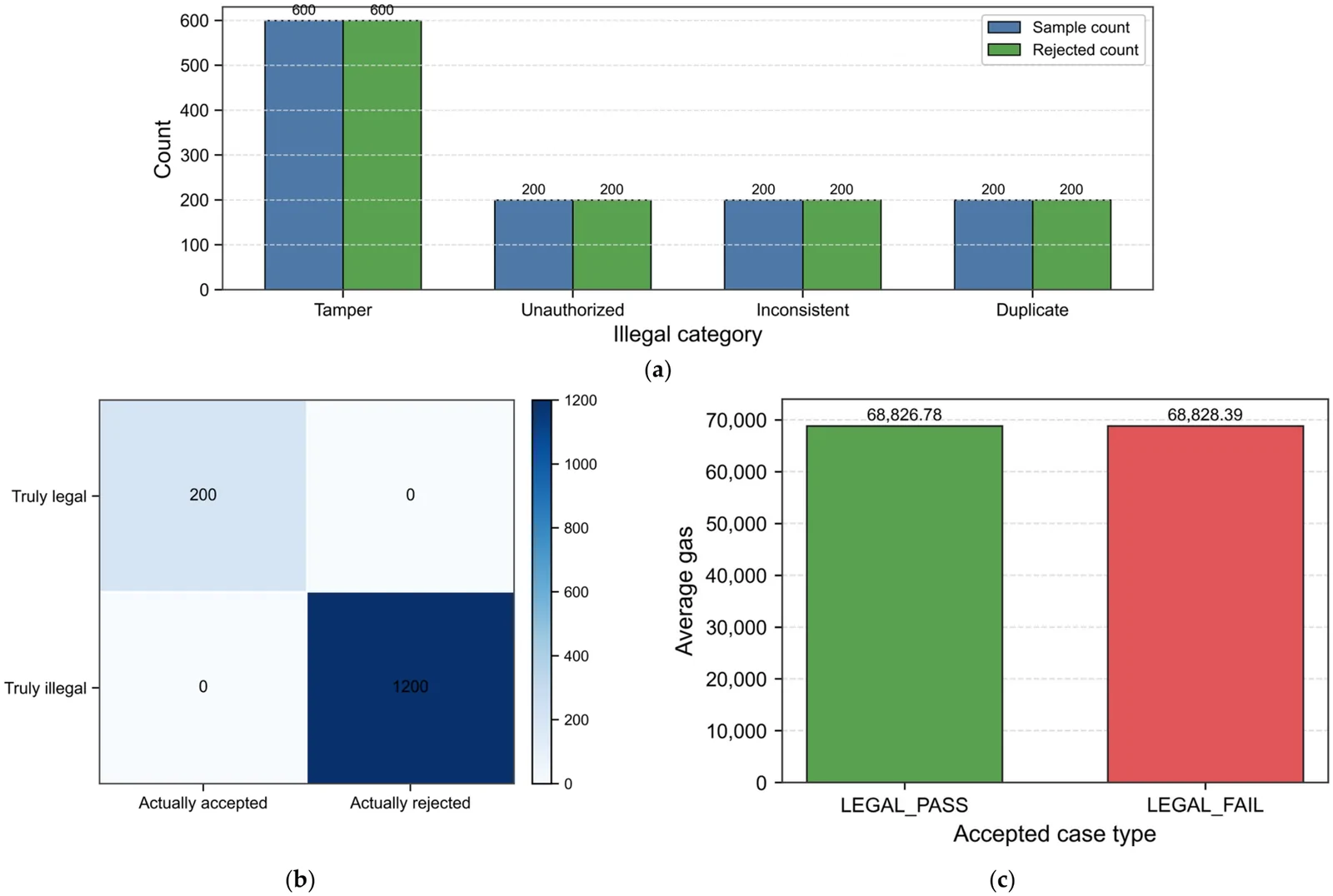
Results of second-track LEGAL/ILLEGAL confirmation and contract-rule enforcement: (**a**) number of submissions and rejected submissions under different ILLEGAL categories; (**b**) determination matrix of LEGAL and ILLEGAL submissions; (**c**) Gas consumption of LEGAL_PASS and LEGAL_FAIL.

To evaluate contract-side enforcement of the second-track confirmation rules, 1400 structured confirmation records were constructed to test signature verification, device authorization, field integrity, result consistency, and duplicate-submission interception. The test set comprises 200 LEGAL submissions, including 100 LEGAL_PASS and 100 LEGAL_FAIL records, and 1200 ILLEGAL submissions covering field tampering, unauthorized devices, result inconsistency, and duplicate submission. The results are shown in Figure 9 and Table 13.

Figure 9a,b and Table 13 show that all 200 LEGAL submissions are accepted according to the predefined contract rules, whereas all 1200 ILLEGAL submissions are rejected. LEGAL_PASS denotes a qualified result submitted by an authorized device and restores the batch from YELLOW to GREEN, whereas LEGAL_FAIL denotes an over-threshold result and updates the batch from YELLOW to RED. The four ILLEGAL categories—Tamper, Unauthorized, Inconsistent, and Duplicate—are all rejected before an unauthorized state transition can occur. These results demonstrate deterministic enforcement of the predefined signature-integrity, device-authorization, result-consistency, and submission-uniqueness checks under the tested cases.

Importantly, the LEGAL/ILLEGAL outcomes reported here describe smart-contract validation of second-track confirmation records rather than analytical classification of AFB1 contamination. Accordingly, rejection of an ILLEGAL submission should not be interpreted as a false-positive/false-negative result of an AFB1 detection method. The experiment evaluates whether structured confirmation data satisfy the predefined state-update rules, not whether the upstream food-hazard measurement itself is analytically correct.

Figure 9c shows that the mean Gas consumption of LEGAL_PASS and LEGAL_FAIL is 68,826.78 and 68,828.39, respectively, with only a negligible difference. The execution costs of signature verification, threshold determination, and state updating were similar for the two confirmation outcomes under the tested conditions. Because second-track confirmatory data are small structured records, they can be submitted directly on-chain without the large storage overhead associated with high-dimensional preliminary screening files.

Overall, the second-track mechanism prevents a YELLOW batch from being released or blocked on the basis of a confirmation that fails the predefined integrity, authorization, consistency, or uniqueness checks. LEGAL_PASS and LEGAL_FAIL drive the expected GREEN and RED state transitions, respectively, whereas ILLEGAL submissions are rejected without changing the pending YELLOW state. These results demonstrate deterministic enforcement of the second-track confirmation rules rather than analytical accuracy of AFB1 detection. The integration of these second-track state transitions with multi-oracle source-batch determination and associated-batch locking is evaluated through the end-to-end workflows in Section 4.5.

### 4.5. End-to-End Workflow Characterization and Besu-QBFT Multi-Node Validation

**Figure 10:**
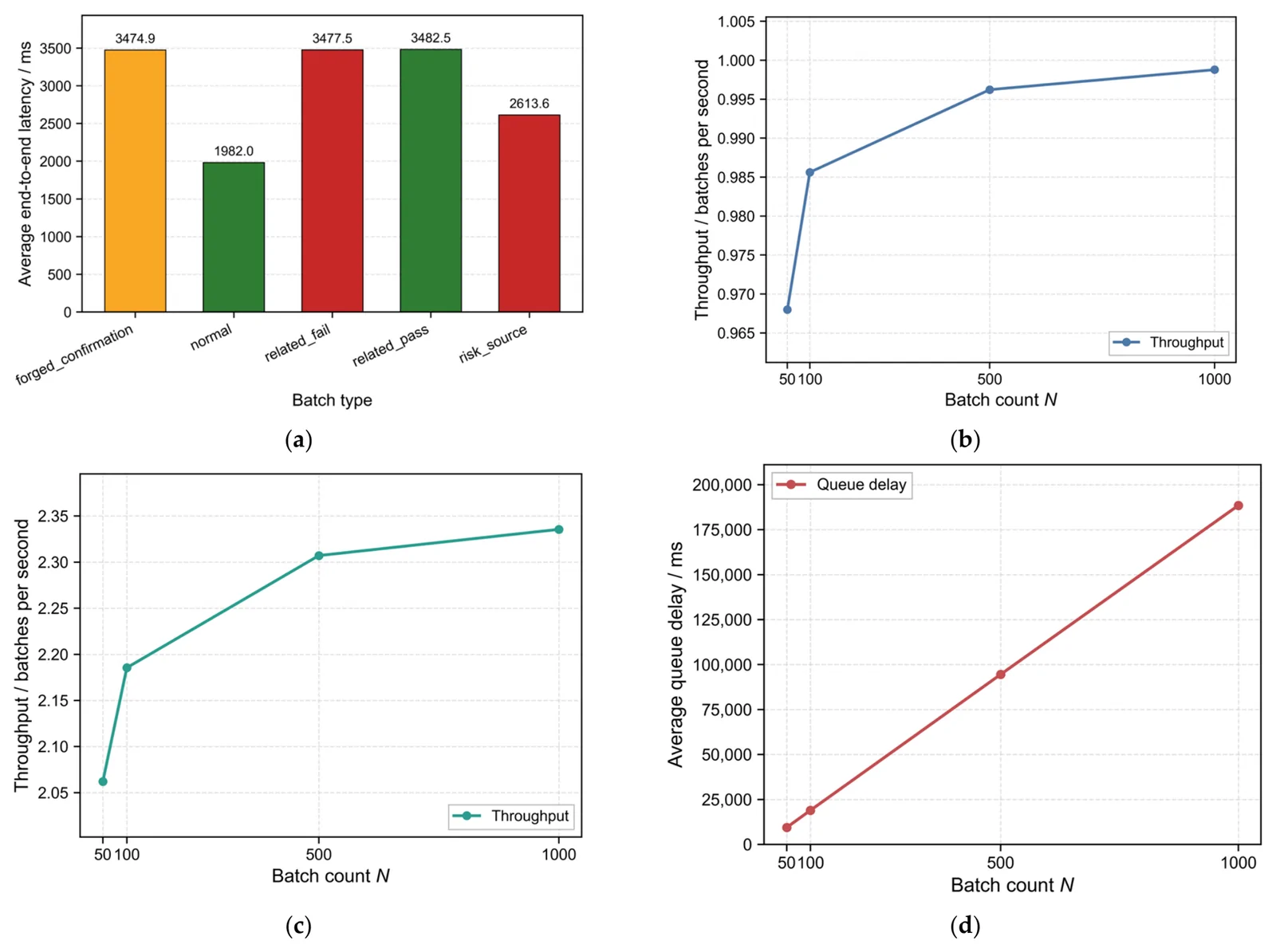
Hardhat-based end-to-end workflow characterization under normal- and high-load conditions: (**a**) mean end-to-end latency of different batch types; (**b**) throughput variation with N under normal load; (**c**) throughput under high load; (**d**) queuing latency variation under high load.

**Figure 11:**
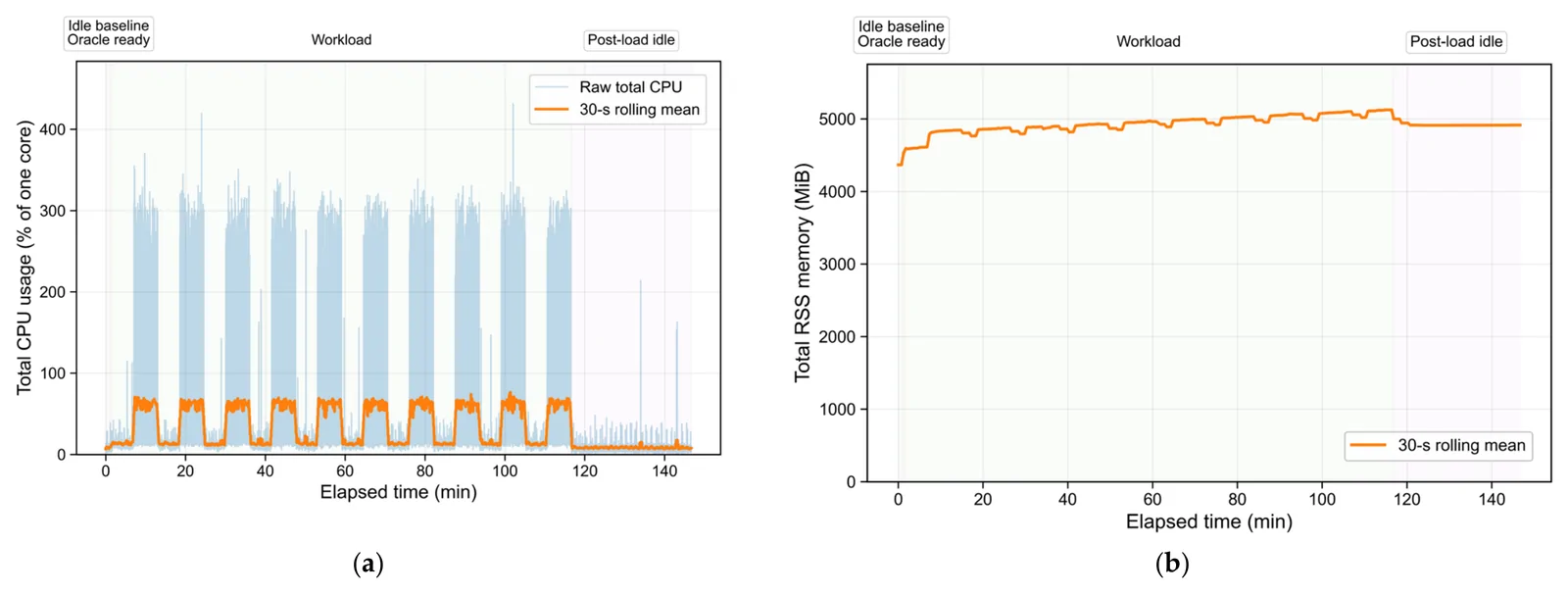
Resource behavior during sustained fixed-contract operation on the four-node Hyperledger Besu deployment using Quorum Byzantine Fault Tolerance (QBFT): (**a**) total central processing unit (CPU) utilization during sustained fixed-contract operation; (**b**) total resident set size (RSS) memory during sustained fixed-contract operation. The shaded background regions indicate the idle-baseline, oracle-ready, workload, and post-load idle stages, as labeled in the plots.

This section presents two complementary system-level evaluations. First, a Hardhat-based controlled workload experiment characterizes end-to-end latency, throughput, queuing behavior, and state-transition execution under normal- and high-load settings. Second, real-file-driven and sustained experiments on the four-node Besu-QBFT network evaluate the complete sequence of multi-oracle source-batch determination, associated-batch locking, second-track confirmation, repeated fixed-contract reuse, resource behavior, and replicated-state consistency. The corresponding results are presented in Figure 10 and Figure 11 and Table 14.

The Hardhat-based controlled workload characterization includes normal- and high-load scenarios. Under the normal-load setting, the input rate is 1 batch/s to characterize workflow execution without sustained task accumulation. Under the high-load setting, the submission rate is increased beyond the observed processing rate to characterize throughput saturation and queuing behavior under the tested configuration. Within this controlled workload experiment, five batch types are evaluated: normal, risk_source, related_pass, related_fail, and ILLEGAL forged-confirmation workflows. The related-pass and related-fail workflows correspond to LEGAL_PASS and LEGAL_FAIL second-track confirmations, respectively, whereas the forged-confirmation workflow contains an ILLEGAL confirmation that is rejected by the contract.

Within the Hardhat-based controlled workload environment, Figure 10a shows that normal workflows have the lowest mean end-to-end latency, approximately 1.98 s. The mean latency of risk-source workflows is approximately 2.61 s because RED state updating and associated-index triggering introduce additional processing. Related-pass, related-fail, and ILLEGAL forged-confirmation workflows exhibit mean latencies of approximately 3.47–3.48 s because they include association locking and second-track processing before the final state is obtained. The resulting GREEN, RED, and YELLOW states are consistent with the predefined state-transition rules; this consistency is interpreted as correct workflow execution rather than an independent measure of food-detection accuracy.

Figure 10b–d show that, at an input rate of 1 batch/s, throughput remains close to the submission rate without evident sustained task accumulation. Under the tested high-load settings, throughput approaches approximately 2.3 batches/s, while mean queuing latency increases from approximately 9.43 s to 188.50 s as the submission rate exceeds the observed processing rate. These results characterize the saturation and queuing behavior of the Hardhat-based controlled workload environment and indicate that task accumulation becomes the principal contributor to additional delay after saturation. They should not be interpreted as Besu-QBFT network throughput or as a production-scale throughput limit.

A separate real-file-driven end-to-end evaluation reused the 57 independent HyperPistachio inputs across five system-level rounds, resulting in 285 workflow executions. Each round preserved the 51 GREEN and six RED source-input composition, yielding 255 GREEN- and 30 RED-source workflows across the five rounds. Each RED-source workflow was configured with two controlled associated batches, one receiving a LEGAL_PASS confirmation and the other a LEGAL_FAIL confirmation, resulting in 60 associated-batch instances across the five rounds. Here, PASS → FAIL and FAIL → PASS denote the submission order of these confirmations for the two different associated batches within the same RED-source workflow; they do not represent sequential state changes of the same batch. The two confirmation orders were balanced across the 30 RED-source workflows, with each order exercised 15 times. The same expected final-state pattern was obtained under both confirmation orders, and the recorded workflow states remained consistent across all four Besu nodes. These 285 workflow executions therefore provide end-to-end evidence for the sequence from multi-oracle source-batch determination to associated-batch locking and second-track confirmation while remaining system-level repetitions rather than additional independent food observations.

To further examine repeated operation on the same Besu-QBFT deployment, the multi-oracle controller and dual-track risk-control contracts remain deployed and are reused while the 57 independent HyperPistachio inputs are processed through the workflow for 10 consecutive rounds. This produces 570 repeated workflows, including 510 GREEN- and 60 RED-source workflows, together with 1710 oracle executions, 4593 on-chain transactions, and 2760 state snapshots. Across the 60 RED-source workflows, the order in which PASS and FAIL confirmations were submitted for controlled associated batches was balanced, with PASS → FAIL and FAIL → PASS each exercised 30 times. The active workload lasts 115.15 min, followed by a 29.98 min observation period without new workflow submissions. The value 570 therefore represents repeated executions of the 57 independent files rather than 570 independent food samples.

Throughout the sustained run, the four Besu nodes maintain mutually consistent workflow states and finish at the same block height of 51,881. The fixed contracts are deployed once and continuously reused across all 10 rounds, indicating that the state-control workflow can operate repeatedly without contract redeployment between batches.

Figure 11a shows the aggregate central processing unit (CPU) behavior during sustained operation. Periodic peaks accompany continuous workflow execution, whereas CPU utilization decreases rapidly after the active workload ends. The mean utilization of all monitored processes is approximately 39.02% of one logical CPU core, indicating that the tested workload does not produce sustained CPU saturation in the current environment.

Figure 11b shows that total RSS memory increases during the active workload and reaches a high level near the end of the run, but does not continue increasing after workload termination. The mean resident set size (RSS) is 4935.64 MiB, with a 95th percentile (P95) of 5106.78 MiB and a maximum of approximately 5138.77 MiB. During the post-load observation period, total RSS decreases and stabilizes at approximately 4.8 GiB. Thus, no unbounded memory-growth pattern is observed within the approximately 115 min active workload and subsequent 30 min observation window. This result does not exclude memory drift or leakage over substantially longer deployment periods.

Overall, the Hardhat-based workload characterization identifies the latency, throughput-saturation, and queuing behavior of the workflow under the tested controlled-load settings. Separately, the Besu-QBFT experiments show that the complete real-file-driven dual-track sequence can execute from multi-oracle source-batch determination through associated-batch locking and second-track confirmation, with consistent final-state outcomes under both tested confirmation orders. The sustained multi-node experiment further shows that the same deployed multi-oracle and dual-track contracts can be repeatedly reused with real-file-driven workflows while maintaining consistent replicated states. The Hardhat workload results and the Besu-QBFT multi-node results therefore serve different validation purposes and are not treated as directly interchangeable performance measurements. The sustained-run results remain limited to the single-host logical multi-node environment described in Section 3.1 and should not be interpreted as cross-institutional WAN or production-deployment performance.

### 4.6. Case Validation of Batch-Level Risk Control Using Pistachio AFB1 Detection Data

To evaluate whether real high-dimensional food-detection files can be incorporated into the proposed batch-level risk-control workflow, the HyperPistachio dataset [33] was used as the real-file input. The case validation was based on 57 independent hyperspectral sample units. Each sample contained a BIL hyperspectral data file, an HDR metadata file, and a PNG preview image, and each BIL file used in the experiment was 90,832,896 B (86.625 MiB). HyperPistachio identifies the target contaminant as aflatoxin B1 (AFB1) and provides concentration annotations in μg/kg [33]. These published concentration labels were used directly as external inputs in this study; the samples were not chemically remeasured, and no new AFB1 concentration values were generated from the hyperspectral images by the proposed system. The experimental input set contains 57 unique sample identifiers, with AFB1 labels ranging from 0.40 to 33.17 μg/kg.

Accordingly, this experiment evaluates the management of existing AFB1 detection results rather than the analytical detection of AFB1 itself. No hyperspectral classification or regression model, analytical assay, or concentration-estimation algorithm was trained, calibrated, or evaluated in this study. Therefore, analytical sensitivity, specificity, or false-positive and false-negative rates for AFB1 detection cannot be derived from these results. The LEGAL/ILLEGAL outcomes reported in Section 4.4 represent smart-contract validation of second-track confirmation records and should not be interpreted as analytical false-positive or false-negative results. This distinction directly bounds the role of the blockchain layer: it records, transfers, verifies, and acts on externally supplied detection information, but does not independently establish the physical correctness of the original measurement.

For reproducible evaluation of the state-transition workflow, the system screening threshold was fixed at 10.0 μg/kg throughout the experiment. This value is treated as an engineering configuration of the prototype rather than an AFB1 regulatory-compliance limit. Codex CXS 193-1995 specifies a maximum limit of 10.0 μg/kg for total aflatoxins in ready-to-eat pistachios [34], whereas the European Union (EU), through Commission Regulation (EU) 2023/915 distinguishes AFB1 from the sum of aflatoxins and specifies maximum levels of 8.0 μg/kg for AFB1 and 10.0 μg/kg for total aflatoxins in pistachios placed on the market for the final consumer or used as food ingredients [35]. Because the HyperPistachio labels used here represent AFB1, the numerical value of 10.0 μg/kg is used only as the predefined system screening boundary and is not interpreted as regulatory equivalence or as an independent food-compliance decision.

**Figure 12:**
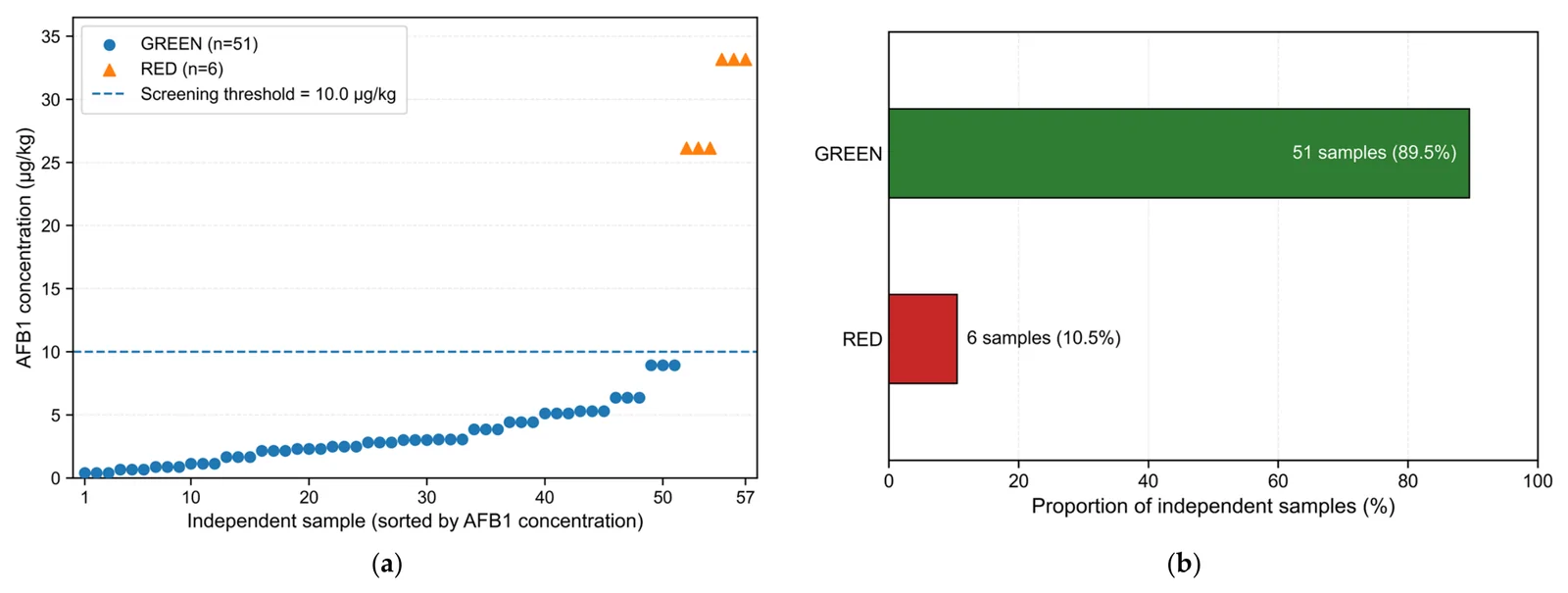
Aflatoxin B1 (AFB1) label distribution and system-state mapping of the 57 independent HyperPistachio samples: (**a**) AFB1 concentration distribution of the 57 independent HyperPistachio samples; (**b**) input-state distribution under the 10.0 μg/kg system screening threshold.

Under this predefined screening rule, 51 of the 57 independent samples were mapped to GREEN inputs, and six were mapped to RED inputs. Figure 12a presents the AFB1 concentration distribution of the 57 samples after sorting by concentration, together with the fixed screening boundary. Figure 12b summarizes the resulting state composition, with GREEN and RED accounting for 89.5% and 10.5% of the independent inputs, respectively. GREEN therefore indicates only that the dataset-provided AFB1 value does not trigger the RED state under the experimental screening rule; it does not certify that the sample is free of other contaminants or that the food is safe in an overall regulatory sense.

The real BIL/HDR files were stored off-chain and referenced through IPFS CIDs, while their externally supplied AFB1 labels provided the structured values required by the state-control workflow. As demonstrated in Section 4.2, the complete 86.625 MiB BIL files can be stored and retrieved in the evaluated multi-Kubo environment without being written directly to the blockchain. The oracle layer therefore serves as the interface for bringing an externally supplied detection result into the contract workflow rather than as an AFB1 detection model. Once a source batch triggers the RED state, the “stage + device + operation identifier” index identifies controlled associated batches and places them in YELLOW pending second-track confirmation. A LEGAL_PASS confirmation releases an associated batch to GREEN, a LEGAL_FAIL confirmation updates it to RED, whereas an ILLEGAL confirmation is rejected and the batch remains YELLOW.

The 57 independent samples define the food-data basis of the AFB1 case, whereas closed-loop behavior is evaluated through repeated system-level executions. As reported in Section 4.5, reuse of these inputs across five end-to-end rounds produced 285 workflows, including 30 RED-source workflows in which PASS → FAIL and FAIL → PASS confirmation orders were each exercised 15 times. The 10-round sustained experiment further produced 570 workflows, including 60 RED-source workflows, with the two confirmation orders each exercised 30 times. These repetitions demonstrate real-file-driven execution of the source-batch determination–associated-batch locking–second-track confirmation loop but do not increase the number of independent food samples beyond 57.

The stage, device, operation, and second-track relationships used for associated-batch control were constructed experimental configurations rather than records collected from an operating food enterprise. Accordingly, the repeated system-level workflows provide evidence regarding execution consistency and system behavior, but should not be interpreted as additional biological observations or as evidence of statistical generalization to wider pistachio populations.

Table 15 summarizes the evidence basis and interpretation boundary of this case. Overall, the HyperPistachio validation demonstrates that real AFB1-labeled high-dimensional detection files can be incorporated into a traceable workflow linking off-chain file access, externally supplied detection results, contract-based state determination, associated-batch locking, and second-track confirmation. The evidence supports the executability of managing existing detection results and translating them into batch-level control actions; it does not demonstrate analytical AFB1 detection accuracy, regulatory compliance of individual samples, or validated applicability to food hazards other than AFB1.

### 4.7. Comparison with Conventional Digital Management Approaches and Practical Implications

To clarify the practical role of the proposed mechanism, Table 16 compares it with centralized databases, laboratory information management systems (LIMS), and conventional food traceability platforms at the architectural level. This is not a direct performance benchmark because the alternative systems were not deployed under identical hardware and workload conditions. Centralized databases and LIMS are generally efficient within a single trusted organization, whereas conventional traceability platforms primarily support product, process, and lot tracking [21]. The proposed mechanism addresses a different requirement: linking detection evidence and externally supplied analytical results to auditable cross-organizational batch-state actions.

Compared with these conventional approaches, the main distinction of the proposed mechanism lies in connecting traceability information directly to batch-risk control. A detection result can trigger a RED source-batch state, identify associated batches through the stage–device–operation relationship, temporarily place them in YELLOW, and subsequently release or block them according to second-track confirmation. Thus, traceability records are used not only for retrospective inquiry but also as inputs to predefined control actions. In this workflow, traceability is provided by the persistent linkage among detection evidence, batch identifiers, and state transitions, while accountability is supported by recording the authorized identities and signed actions responsible for each state-changing event.

The additional auditability of the proposed mechanism is achieved at a higher operational cost. In Section 4.2, three-oracle arbitration increases total submitted Gas by approximately 74.3% compared with the single-oracle mode, while Section 4.3 shows that associated-batch state-update Gas increases with fanout. Therefore, the mechanism is not intended to replace centralized databases or LIMS when one trusted administrator is sufficient. In practical deployment, existing LIMS and enterprise traceability systems can retain their operational data, while the blockchain layer records selected hashes, CIDs, signed results, batch identifiers, and control events that require shared verification.

The need for such coordination is reflected in current food-safety traceability practice. The U.S. Food and Drug Administration (FDA) Food Traceability Rule links key data elements to traceability lots at specified critical tracking events [36], while the EU Alert and Cooperation Network recorded 10,490 notifications in 2025 and reported cross-border incidents requiring rapid source identification and targeted control [37]. These examples do not imply that blockchain prevents contamination; rather, they illustrate the practical value of linking analytical evidence, lot identity, and control actions. Within predefined organizational procedures, such auditable linkage can support regulatory coordination, rapid source identification, targeted batch control, and transparent responsibility tracing. Accordingly, the proposed mechanism is positioned as a complementary coordination layer for multi-party food-safety management rather than a universal replacement for existing digital systems.

## 5. Conclusions

This study developed a blockchain-based dual-track mechanism for trusted circulation of food-safety detection data and batch-level risk control. The mechanism combines IPFS-CID anchoring for high-dimensional files, two-of-three multi-oracle arbitration for externally supplied analytical results, a stage–device–operation inverted index for associated-batch localization, and signature-based second-track confirmation within a unified GREEN/YELLOW/RED state-control workflow. Its contribution lies in connecting detection evidence with auditable downstream batch actions rather than in proposing a new analytical method for contaminant detection.

The experiments demonstrate the main technical characteristics and trade-offs of the proposed mechanism. Fifty-seven independent HyperPistachio samples with 86.625 MiB BIL files were incorporated into the on-chain/off-chain workflow. Direct-on-chain storage reached the tested transaction-capacity boundary between 40 and 44 KiB, whereas IPFS-CID kept the on-chain representation independent of the original file size. Under normal conditions, the three-oracle mode showed no clear increase in final arbitration latency relative to the single-oracle mode, although total submitted Gas increased by approximately 74.3%. The two-of-three mechanism tolerated a single unavailable, incorrect, delayed, or evidence-failing oracle when two consistent reports remained, while majority collusion remained an explicit trust boundary. As associated-batch fanout increased from 1 to 40, index-query latency increased from 13.06 to 16.78 ms, with the main additional cost arising from on-chain state updates. During the 115.15 min sustained experiment, 570 repeated workflows were executed; PASS → FAIL and FAIL → PASS confirmation orders were each exercised 30 times among the 60 RED-source workflows, and all four Besu nodes maintained consistent recorded states.

The empirical evidence is limited to the management of externally supplied AFB1 results and does not establish analytical AFB1 detection accuracy, regulatory compliance of individual samples, or validated applicability to other contaminants. From a practical perspective, the proposed mechanism is therefore better positioned as a complementary coordination layer rather than a replacement for existing LIMS, centralized databases, or traceability platforms. Such systems can retain laboratory and operational data, while selected CIDs, signed results, batch identifiers, and control events are shared through the blockchain layer when cross-organizational verification is required. The same architectural principle may be adapted to other food-safety detection data only after hazard-specific analytical inputs, thresholds, association rules, and operational requirements have been independently validated.

## 6. Limitations and Future Work

This study has two main limitations. First, the empirical food-data validation is restricted to 57 independent HyperPistachio samples and externally supplied AFB1 concentration labels. The analytical concentrations were not remeasured in this study, and no AFB1 detection or quantification model was trained or evaluated. Moreover, the stage–device–operation relationships and second-track confirmation scenarios were constructed experimental configurations rather than records collected from an operating food enterprise. The 285 end-to-end workflow executions and 570 sustained-operation workflows are repeated system-level executions derived from the same 57 independent inputs and therefore do not constitute additional biological observations. The results therefore support the executability of the proposed data-management and batch-control workflow for the evaluated AFB1 case, but do not establish statistical generalization to wider pistachio populations or applicability to other food contaminants.

Second, the system-level evaluations were conducted in controlled local prototype environments. The normal- and high-load workload characterization used Hardhat, whereas the Besu-QBFT experiments used a single-host logical multi-node deployment. Consequently, the results do not represent cross-institutional WAN performance or long-term production deployment. In addition, two-of-three arbitration reduces dependence on one oracle but cannot prevent an incorrect result when a majority of authorized oracles provide the same incorrect value–evidence pair. Consequently, organizational independence, oracle governance, and responsibility for upstream analytical results remain important deployment considerations.

Future work should focus on interoperability, broader food-data validation, and cross-organizational deployment requirements. First, validation should be extended to larger and more diverse independently collected hyperspectral datasets, including additional food samples, batches, acquisition conditions, and, where available, independently established analytical reference values. Such data would allow the workflow to be evaluated across a broader range of real detection files and externally supplied analytical inputs without treating repeated system executions as additional biological evidence. Standardized semantic representations and trusted exchange specifications for detection evidence, thresholds, device identities, operation records, and regulatory entities could facilitate integration with existing LIMS and traceability systems [38,39]. Cross-institutional deployment should also consider organizationally independent oracle operators, clearer governance and responsibility-allocation mechanisms, and more robust result-aggregation strategies [40,41]. Future validation should also incorporate independently collected operational batch, device-use, and confirmation records from multi-enterprise food-supply scenarios. These directions would support more reliable multi-party collaboration while preserving the distinction between trusted management of analytical results and verification of the analytical measurements themselves.

## Figures and Tables

**Table 1:** Definition of batch states.

State	Meaning	Processing Rule
GREEN	The current batch state does not trigger the risk-control rule under the supplied detection result.	The prototype workflow permits the batch to proceed to the next supply-chain stage.
YELLOW	The batch is matched by the hash-based inverted index and has an associated risk due to shared equipment.	The prototype workflow suspends progression pending second-track confirmation.
RED	The batch has been determined as a risk batch by the left track or confirmed as risky by the second track.	The prototype workflow blocks further circulation.

**Table 2:** Batch state transition rules.

Current State	Triggering Event	Next State	Meaning
GREEN	$e_{\mathrm{safe}}$	GREEN	The supplied result does not trigger the configured risk-control threshold, and the prototype workflow permits progression.
GREEN	$e_{\mathrm{risk}}$	RED	The current batch is identified as the risk source.
GREEN	$e_{\mathrm{lock}}$	YELLOW	The batch is locked as an associated batch and waits for confirmation.
YELLOW	$e_{\mathrm{pass}}$	GREEN	The second-track confirmation is passed, and circulation is restored.
YELLOW	$e_{\mathrm{fail}}$	RED	The second-track confirmation fails, and circulation is prohibited.
YELLOW	$e_{\mathrm{invalid}}$	YELLOW	The illegal confirmation is rejected, and the batch remains pending confirmation.
RED	Any event	RED	A risk batch cannot be restored to a circulating state.

**Table 3:** Verification rules for second-track signature-based confirmation.

Verification Item	Judgment Condition	Processing When the Condition Is Not Satisfied
Signature integrity	$\mathrm{Verify}(pk_{d_{i}},M_{i},\sigma_{i})=\mathrm{true}$	Reject the state update
Device authorization	$d_{i}\in D_{\mathrm{auth}}$	Reject the state update
Result consistency	$\mathrm{Consistent}(C_{i})=\mathrm{true}$	Reject the state update
Duplicate submission check	$\mathrm{Confirmed}(b_{i})=\mathrm{false}$	Reject the state update
State precondition	$\mathrm{State}(b_{i})=\mathrm{YELLOW}$	Reject the state update

**Table 4:** Experimental environment and tool configuration.

Module	Tool or Platform	Function
Operating system	Ubuntu 22.04.5 LTS	Experimental runtime environment
Smart contract	Solidity 0.8.20	Implementation of contract logic
Permissioned blockchain	Hyperledger Besu 25.11.0; four QBFT validators	Multi-node on-chain execution and replicated-state validation
Off-chain file storage	Three separate Kubo/IPFS 0.41.0 repositories	Detection-file storage, replication, and CID-based retrieval
Oracle program	Three logical oracle services with 2-of-3 arbitration	CID-based file retrieval and consistent result/evidence submission
Contract development and testing	Hardhat 2.28.6	Contract compilation, unit testing, state-machine validation, and controlled workload characterization
Data analysis	Python 3.11.5, pandas 1.3.5; Matplotlib 3.5.1	Result statistics and figure generation

**Table 5:** Core smart contract functions.

Function	Description
createBatch()	Creates a food batch and initializes its state.
submitCID()	Submits the IPFS CID and triggers an oracle listening event.
applyOracleResult()	Applies the 2-of-3 arbitrated oracle result and enables threshold determination and batch-state updating.
registerEquipmentIndex()	Registers the device usage index key of a batch.
lockRelatedBatches()	Locks associated batches according to the hash-based inverted index.
submitConfirmation()	Submits the second-track confirmation result and device signature.
getBatchStatus()	Queries the current state of a batch.

**Table 6:** Data sources and experimental purposes.

Dataset	Source/Construction Method	Sample Size	Main Fields	Experimental Purpose
HyperPistachio real hyperspectral files	Public HyperPistachio dataset	57 independent samples; 57 BIL, 57 HDR, and 57 PNG files	sampleId, BIL/HDR files, CID, file hash	To verify real high-dimensional file storage, CID-based retrieval, and on-chain/off-chain workflow execution.
AFB1 concentration labels	Labels provided by HyperPistachio	57 labels corresponding to 57 independent samples; 51 GREEN and 6 RED inputs under the 10.0 μg/kg system screening threshold	sampleId, AFB1 concentration, valueScaled, state	To provide predefined detection-result inputs for oracle arbitration and contract-based state determination.
Device-sharing relationship data	Scenario-based construction	Controlled stage–device–operation relationships	batchId, stage, deviceId, jobId	To verify associated-batch locking and fanout scalability of the hash-based inverted index.
Second-track confirmatory data	Constructed LEGAL and ILLEGAL confirmation scenarios	LEGAL_PASS, LEGAL_FAIL, and ILLEGAL submissions	value, threshold, result, deviceId, signature	To verify signature integrity, device authorization, result consistency, state recovery/blocking, and ILLEGAL-submission rejection.
Repeated workflow data	Repeated execution using the 57 independent real samples	Five rounds and 285 workflows for end-to-end dual-track evaluation; 10 rounds and 570 workflows for sustained operation	sampleId, oracle result, batch state, transaction and state records	To evaluate closed-loop state transitions, second-track confirmation-order behavior, repeated execution, and sustained multi-node operation; workflow counts are not treated as independent food samples.

**Table 7:** Semantic sources and usage boundaries of experimental data.

Data	Source/Basis	Usage in This Study	Boundary Clarification
Real HyperPistachio files	Public hyperspectral dataset	Used as real high-dimensional inputs for IPFS storage, CID access, and oracle retrieval.	The 57 samples are the independent real-file inputs; repeated workflows do not increase the number of independent samples.
AFB1 concentration labels	Values supplied by the HyperPistachio dataset	Used as predefined detection values for oracle result formation and smart-contract state determination.	The concentrations are not measured by this study, and GREEN/RED are system states rather than independent regulatory or overall food-safety conclusions.
Device and operation relationships	Scenario-based construction	Used to evaluate associated-batch locking and second-track state control.	These relationships are controlled experimental configurations and do not represent records collected from an operating food enterprise.
Second-track confirmation data	Scenario-based construction	Used to verify PASS/FAIL confirmation and rejection of abnormal submissions.	These records validate system-control logic rather than independent food-testing outcomes.

**Table 8:** Experimental design and evaluation metrics.

Experimental Module	Corresponding Result Subsection	Experimental Objective	Main Metrics
IPFS-CID notarization, real-file access, and oracle processing	4.2	To verify the feasibility and execution efficiency of incorporating high-dimensional detection files into the on-chain state determination process through off-chain storage, CID anchoring, and oracle processing, and to evaluate the performance and fault boundary of single- and multi-oracle modes.	File integrity, IPFS add/retrieval time, Gas consumption, transaction-capacity boundary, oracle decision latency, O0/O3 performance difference, and fault-condition outcomes
Hash-based inverted-index-associated locking experiment	4.3	To verify whether the hash-based inverted index based on “stage + device + operation identifier” can selectively identify and lock associated batches defined by the stage–device–operation relationship and reduce excessive blocking, and to evaluate state consistency and execution overhead as associated-batch fanout increases.	Number of locked batches, locking reduction rate, fanout, Gas consumption, and index-query time
Second-track signature-based confirmation experiment	4.4	To evaluate contract-side enforcement of signature integrity, device authorization, result consistency, and duplicate-submission checks for structured confirmation records.	Acceptance of LEGAL_PASS and LEGAL_FAIL, rejection of ILLEGAL tampering, unauthorized, inconsistent, and duplicate submissions, state-update results, and Gas consumption
End-to-end workflow characterization and multi-node validation	4.5	To characterize end-to-end workflow latency, throughput, and queuing behavior under controlled normal- and high-load conditions, and separately to evaluate complete real-file-driven dual-track execution, confirmation-order behavior, repeated fixed-contract reuse, resource behavior, and replicated-state consistency in the four-node Besu-QBFT environment.	End-to-end latency, throughput, and queuing latency under controlled workload conditions; confirmation-order outcomes, four-node state consistency, workflow count, oracle executions, on-chain transactions, central processing unit (CPU), resident set size (RSS), and sustained-run duration in the Besu-QBFT experiments
HyperPistachio real-file-driven case validation	4.6	To characterize the real-file and AFB1-data basis of the case and clarify how the 57 independent food inputs relate to the repeated system-level workflow evidence.	Independent sample count, BIL file size, AFB1 input-state distribution, associated-batch states, file retrieval performance, and workflow-state consistency

**Table 9:** Key results for real-file access and on-chain storage.

Metric	Result
Independent real BIL files	57
BIL file size	90,832,896 B (86.625 MiB)
Mean add time in Kubo 1	402.916 ms
Mean retrieval time in Kubo 1	414.038 ms
Mean retrieval time in Kubo 2	423.185 ms
Mean retrieval time in Kubo 3	434.753 ms
Maximum successful Direct-on-chain payload	40 KiB
First failed Direct-on-chain payload	44 KiB
Gas consumption at 40 KiB	29,245,745
Block Gas limit	30,000,000
Mean Hash-only Gas	≈140,743
Mean IPFS-CID Gas	≈207,921
Projected Gas for one real BIL file	≈6.45 × 10^10^
Mean add time for an 86.625 MiB unique object	435.955 ms
Mean parallel replication time for an 86.625 MiB object	563.928 ms
Mean retrieval time for an 86.625 MiB unique object	455.108 ms

**Table 10:** Oracle fault conditions and two-of-three arbitration outcomes.

Fault Condition	Mode	Expected Outcome	Actual Outcome	Repetitions	Mean Finalization Time/ms
Normal operation	O3	Correct finalization	Correct finalization	5	1902.05
Single oracle offline	O0	PENDING	PENDING	5	—
Single oracle offline	O3	Correct finalization	Correct finalization	5	1965.66
Single oracle returns an incorrect result	O0	Incorrect finalization	Incorrect finalization	5	1815.66
Single oracle returns an incorrect result	O3	Correct finalization	Correct finalization	5	3817.06
Single oracle delayed	O0	Correct finalization	Correct finalization	5	5892.55
Single oracle delayed	O3	Correct finalization	Correct finalization	5	1821.44
Single evidence-validation failure	O0	PENDING	PENDING	5	—
Single evidence-validation failure	O3	Correct finalization	Correct finalization	5	1965.54
Two oracles offline	O3	PENDING	PENDING	5	—
Two oracles collude with an incorrect result	O3	Incorrect finalization	Incorrect finalization	5	1846.37
Unauthorized replay	O3	Correct finalization	Correct finalization	5	1840.77
Split results without a 2-of-3 majority	O3	PENDING	PENDING	5	—

**Table 11:** Mean number of locked batches under different locking strategies.

Number of Batches N	Baseline A	Baseline B	Baseline C	Proposed
100	99.00	9.89	54.10	3.45
500	499.00	49.89	255.22	15.74
1000	999.00	100.34	497.93	29.93
5000	4999.00	501.22	2467.81	148.07

**Table 12:** Locking reduction under different device-sharing ratios.

Device-Sharing Ratio ρ	Reduction Relative to Baseline A	Reduction Relative to Baseline B	Reduction Relative to Baseline C	Number of Locked Batches by Proposed
0.10	96.96%	69.31%	94.15%	48.81
0.30	96.95%	68.54%	94.23%	48.49
0.50	96.65%	64.93%	94.13%	50.58

**Table 13:** Contract responses to second-track LEGAL and ILLEGAL submissions.

Submission Category	Number of Submissions	Expected Contract Response	Actual Contract Response	Consistent with Expectation
LEGAL_PASS	100	Accepted, YELLOW → GREEN	Accepted, GREEN	Yes
LEGAL_FAIL	100	Accepted, YELLOW → RED	Accepted, RED	Yes
Tamper	600	Rejected	Rejected	Yes
Unauthorized	200	Rejected	Rejected	Yes
Inconsistent	200	Rejected	Rejected	Yes
Duplicate	200	Rejected	Rejected	Yes

**Table 14:** Summary of sustained fixed-contract operation in the Besu-QBFT environment.

Metric	Result	Interpretation
Independent real files	57	HyperPistachio real-file inputs
Execution rounds	10	Each real file reused across 10 workflow rounds
Total workflows	570	57 × 10; not 570 independent food samples
GREEN/RED-source workflows	510/60	Final states consistent with predefined workflow rules
Second-track confirmation orders	PASS → FAIL: 30; FAIL → PASS: 30	Balanced order of PASS and FAIL submissions for controlled associated batches across the 60 RED-source workflows
Oracle executions	1710	Three oracles participate in each workflow
Total on-chain transactions	4593	Coordinator and oracle-report transactions
State snapshots	2760	Used for multi-node state verification
Active workload duration	115.15 min	Continuous workflow execution
Post-load observation	29.98 min	No new workflow submissions
Mean CPU utilization	39.02% of one core	All monitored processes
Mean RSS	4935.64 MiB	All monitored processes
RSS P95	5106.78 MiB	Sustained-run high-range memory usage
Maximum RSS	5138.77 MiB	Maximum observed value
Final Besu block heights	51,881/51,881/51,881/51,881	Final height difference = 0
Four-node state agreement	100%	All recorded workflow-state checks agree

**Table 15:** Evidence basis and interpretation boundary of the HyperPistachio case.

Item	Result/Configuration	Interpretation
Independent real samples	57	Independent HyperPistachio inputs used in this case
Unique sample identifiers	57	No repeated workflow is counted as an additional independent sample
Target analyte	AFB1	Based on the HyperPistachio dataset definition [33]
File composition	BIL + HDR + PNG per sample	Real hyperspectral-file input
BIL file size	90,832,896 B (86.625 MiB)	Real high-dimensional file size
AFB1 concentration source	Dataset-provided concentration annotations	No chemical remeasurement was performed in this study
AFB1 concentration range	0.40–33.17 μg/kg	Range represented by the 57 experimental inputs
Represented concentration levels	19	Distinct concentration levels among the 57 experimental inputs
System screening threshold	10.0 μg/kg	Prototype state-control threshold; not an AFB1 regulatory limit
GREEN inputs	51 (89.5%)	Did not trigger RED under the predefined system rule
RED inputs	6 (10.5%)	Triggered RED under the predefined system rule
Analytical AFB1 false-positive/false-negative rates	Not evaluated	No AFB1 detection or prediction model was evaluated
Associated-batch relationships	Controlled stage–device–operation configurations	Not observations collected from an operating food enterprise
End-to-end workflow repetitions	285 workflows across five rounds	System-level execution of the complete dual-track sequence; not additional independent food samples
Second-track confirmation-order cases	PASS → FAIL: 15; FAIL → PASS: 15	Balanced order of PASS and FAIL submissions for controlled associated batches during end-to-end evaluation
Sustained workflows	570 workflows across 10 rounds	Repeated fixed-contract execution; not additional independent biological samples
Sustained confirmation-order cases	PASS → FAIL: 30; FAIL → PASS: 30	Repeated second-track execution during the 115.15 min sustained workload

**Table 16:** Architecture-level comparison with conventional digital food-management approaches.

Dimension	Centralized Database	LIMS	Conventional Traceability Platform	Proposed Mechanism
Primary role	General data storage and business processing	Laboratory sample, test, and result management	Product and lot traceability	Detection-data management and batch-risk control
Trust model	Single administrator	Laboratory/institutional authority	Platform operator or participating enterprises	Permissioned multi-node governance
Large detection files	Depends on storage design	Supported according to implementation	Usually not the primary function	Off-chain storage with CID/hash anchoring
Cross-organizational auditability	Depends on access control and logs	Depends on institutional configuration	Depends on platform architecture	Replicated ledger and auditable state transitions
Detection-result-driven control	Application-specific	Mainly laboratory-workflow oriented	Usually traceability-oriented	GREEN/YELLOW/RED state control with associated-batch confirmation
Cost and scalability	Generally favorable within one administrative domain	Favorable for laboratory workflows	Platform-dependent	Additional blockchain, oracle, and state-write overhead
Best-fit scenario	Single-owner information management	Laboratory operations	Routine product and lot tracing	Multi-party detection-result sharing and auditable risk coordination

## Data Availability

The HyperPistachio dataset used in this study is publicly available from Zenodo, as cited in Reference [33]. Additional derived experimental records and implementation materials are available from the corresponding author upon reasonable request, as they are currently being curated as part of ongoing follow-up research and have not yet been publicly released.
